# Synergistic Adhesion and Shape Deformation in Nanowire‐Structured Liquid Crystal Elastomers

**DOI:** 10.1002/adma.202414695

**Published:** 2025-01-19

**Authors:** Robert L. Dupont, Yang Xu, Angana Borbora, Xinyu Wang, Fatemeh Azadi, Kaden Havener, Broderick Lewis, Weichen Deng, Benjamin W. Tan, Shucong Li, Rui Zhang, Yuxing Yao, Uttam Manna, Xiaoguang Wang

**Affiliations:** ^1^ William G. Lowrie Department of Chemical and Biomolecular Engineering The Ohio State University Columbus OH 43210 USA; ^2^ School of Materials Science and Engineering Tongji University Shanghai 201804 China; ^3^ Department of Chemistry Indian Institute of Technology Guwahati Assam 781039 India; ^4^ Department of Physics The Hong Kong University of Science and Technology Clear Water Bay Kowloon Hong Kong SAR China; ^5^ School of Materials Science and Engineering Georgia Institute of Technology Atlanta Georgia 30332 USA; ^6^ Department of Chemical and Biological Engineering The Hong Kong University of Science and Technology Clear Water Bay Kowloon Hong Kong SAR China; ^7^ Division of Chemistry and Chemical Engineering California Institute of Technology Pasadena CA 91125 USA; ^8^ Centre for Nanotechnology Indian Institute of Technology Guwahati Assam 781039 India; ^9^ Jyoti and Bhupat Mehta School of Health Science & Technology Indian Institute of Technology Guwahati Assam 781039 India; ^10^ Sustainability Institute The Ohio State University Columbus OH 43210 USA

**Keywords:** adhesion, liquid crystal elastomers, nanowire structures, stimuli‐responsive materials, superwettability

## Abstract

Nature provides many examples of the benefits of nanoscopic surface structures in areas of adhesion and antifouling. Herein, the design, fabrication, and characterization of liquid crystal elastomer (LCE) films are presented with nanowire surface structures that exhibit tunable stimuli‐responsive deformations and enhanced adhesion properties. The LCE films are shown to curl toward the side with the nanowires when stimulated by heat or organic solvent vapors. In contrast, when a droplet of the same solvent is placed on the film, it curls away from the nanowire side due to nanowire‐induced capillary forces that cause unequal swelling. This characteristic curling deformation is shown to be reversible and can be optimized to match curved substrates, maximizing adhesive shear forces. By using chemical modification, the LCE nanowire films can be given underwater superoleophobicity, enabling oil repellency under a range of harsh conditions. This is combined with the nanowire‐induced frictional asymmetry and the reversible shape deformation to create an underwater droplet mixing robot, capable of performing chemical reactions in aqueous environments. These findings demonstrate the potential of nanowire‐augmented LCE films for advanced applications in soft robotics, adaptive adhesion, and easy chemical modification, with implications for designing responsive materials that integrate mechanical flexibility with surface functionality.

## Graphical abstract

Liquid crystal elastomer films with dense nanowire arrays exhibit synergistic adhesion and shape‐changing properties. They curl toward the nanowire side under heat or chemical vapors but away from it with liquid droplets. Chemical modifications make them superoleophobic underwater, enabling precise droplet manipulation. This biomimetic design integrates enhanced adhesion with stimuli‐responsive deformation, with promising applications in soft robotics and adaptive surfaces.

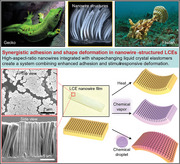

## 1 Introduction

Nature has long inspired the design of advanced materials, particularly through biomimicry, where replicating biological structures leads to enhanced functionalities.^[^ ^1^ ^]^ Notable examples include the antifouling surfaces inspired by filefish scales^[^ ^2^ ^]^ and lotus leaves,^[^ ^3^ ^]^ the surface actuation mechanisms observed in shark skin,^[^ ^4^ ^]^ the anisotropic wettability of filefish, and the remarkable adhesive structures of gecko feet.^[^ ^5^ ^]^ A common feature across these systems is the presence of hierarchical micro‐ and nanostructures that significantly enhance or alter surface interactions.^[^ ^6^ ^]^ For instance, the microscopic setae on gecko feet, ≈90 µm long and 10 µm in diameter, end in hundreds of even smaller spatula‐shaped structures, each ≈20 µm long and 0.2 µm in diameter, dramatically increasing the surface area and enabling strong surface adhesion via van der Waals forces.^[^ ^5^, ^7^ ^]^ Similarly, the microtextured, hook‐like arrays on filefish provide unique antifouling capabilities in oil‐contaminated environments. These well‐ordered surface structures, including nanowires with their high surface area,^[^ ^5e^ ^]^ quantum confinement effects, and enhanced mechanical strength, are critical in advanced technologies, including nanoelectronics,^[^ ^8^ ^]^ photonics,^[^ ^9^ ^]^ catalysts,^[^ ^10^ ^]^ and biomedical devices.^[^ ^11^ ^]^ More importantly, living organisms can actively adjust these nanoscale structures in response to external stimuli, a capability that synthetic materials have yet to fully replicate. For instance, the cytoskeleton in eukaryotic cells demonstrates remarkable dynamic reconfiguration at the nanoscale level. Microtubules, actin filaments, and intermediate filaments—all nanoscale structures—constantly undergo assembly and disassembly in response to various cellular signals and environmental cues.^[^ ^12^ ^]^ This dynamic behavior enables cells to rapidly change shape, divide, and migrate.^[^ ^13^ ^]^ While a precise control over the dimensions and surface characteristics of nanowires has been achieved in synthetic systems, a significant challenge remains in developing materials that can dynamically respond to external stimuli while maintaining the structural integrity and functionality of the nanoscale features observed in these biological systems.

Shape‐changing polymers, particularly liquid crystal elastomers (LCEs), have gained considerable attention due to their unique ability to undergo reversible deformations in response to external stimuli such as temperature,^[^ ^14^ ^]^ light,^[^ ^15^ ^]^ and chemicals.^[^ ^16^ ^]^ LCEs combine the anisotropic properties of liquid crystals (LCs) with the elasticity of polymers, enabling them to exhibit significant shape changes while retaining structural integrity.^[^ ^17^ ^]^ This makes them promising candidates for applications in soft robotics,^[^ ^18^ ^]^ adaptive surfaces,^[^ ^19^ ^]^ and sensors,^[^ ^20^ ^]^ where a precise control over material deformation is crucial. Moreover, the molecular alignment within LCEs allows for a high degree of programmability in their mechanical response, opening avenues for the creation of complex, dynamic structures that can mimic natural movements.^[^ ^21^ ^]^ Despite the potential to unlock new possibilities for designing multifunctional materials that combine the dynamic properties of LCEs with the enhanced surface characteristics of nanostructures, the incorporation of LCEs into such forms, including nanowire arrays integrated with LCE films, remains relatively unexplored. Previous research on LCEs and structured surfaces has been limited in scope, focusing either on LCE‐based structures with microscale dimensions and low aspect ratios, which fail to fully exploit nature‐inspired nanoscale structures,^[^ ^22^ ^]^ or on passive materials with nanoscale structures that exhibit enhanced adhesion but lack stimuli‐responsive shape‐changing capabilities.^[^ ^5^, ^23^ ^]^ These approaches have fallen short in combining the advantages of nanoscale structures with the dynamic responsiveness needed for advanced biomimetic applications, such as the ability to adapt to various surface geometries.

In this work, we present a templated synthesis method to fabricate LCE films with densely packed nanowire arrays on one surface, closely resembling natural structures such as those found on gecko feet.^[^ ^5d^ ^]^ These LCE nanowires, typically measuring 10 µm in length and 400 nm in diameter, are integrally connected to the underlying LCE film, forming a cohesive, hierarchical structure. The similarity in scale to natural nanostructures allows these LCE films to mimic the functional advantages observed in biological systems. These include both advanced adhesive capabilities and underwater superoleophobicity. Additionally, the LCE nanowire films exhibit a sensitivity to temperature and chemical stimuli, curling in response to both. The films also respond differently based on the state of the applied chemical which provides an extra level of control and a decoupling of the directionality of deformation which is typically unseen in many material systems. When exposed to solvent vapor, the films curl toward the nanowire‐covered side of the film, while direct contact with liquid droplets of the same solvent causes the film to curl in the opposite direction. Furthermore, chemical modification of the single‐composition material system further enhances its versatility, rendering the films superoleophobic underwater and providing antifouling capabilities. Combined with the stimuli‐responsive deformations, this allows for controlled manipulation of reactant droplets in aqueous environments. Overall, our work represents a significant advancement in the integration of LCEs with bioinspired nanostructures, offering new possibilities for creating responsive materials that combine the dynamic properties of LCEs with enhanced surface characteristics.

## 2 Results and Discussion

**Figure 1:**
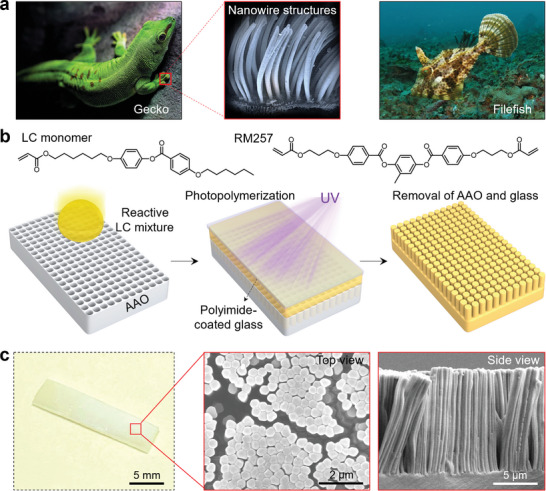
Creation of nanowires on LCE films. a) Photographs of nano/micro‐wire array structures on gecko feet and filefish skin. The typical lengths of the arrays are 30–100 µm for gecko feet and 300–400 µm for fish skin. Photo credit: The gecko and fish images are royalty‐free images from Pixabay company. b) Molecular structure of the LC monomer and crosslinker RM257 used in this work, and the synthesis route for densely packed nanowire arrays on the surface of the LCE films. c) A representative photograph of an LCE nanowire film and the corresponding SEM images of the nanowires on the surface of the film.

Inspired by the nanowire‐like structures of gecko feet and filefish skin (**Figure** **1**a), we fabricated densely packed LCE nanowire arrays on the surface of LCE films. As schemed in Figure 1b, a mixture of a reactive LC monomer and a crosslinker, RM257, was spread on an anodized aluminum oxide (AAO) template (Figure S1, Supporting Information), and was allowed to rest for 10 min at an elevated temperature to infiltrate the mixture into the nanovoids. A polyimide‐coated glass slide was then placed on top, followed by UV exposure to initiate photopolymerization. After removing the AAO template and glass slide, an LCE film with an array of densely packed nanowire arrays on one surface was obtained. The AAO template was etched away using an acidic aqueous solution, and the LCE film was rinsed with water and ethanol to remove leftover acid. Figure 1c shows that these nanowires create a matte texture on the top surface of the LCE film, contrasting with the otherwise shiny base film. Additionally, the nanowires were observed creating bundles on the surface of the film. These bundles were stable under curling conditions as well as after many heating cycles, as shown in Figure S2 (Supporting Information). SEM images reveal the nanowire's dimensions—10 µm in length and 400 nm in diameter—similar to the natural structures found on gecko feet or filefish skin.^[^ ^5^ ^]^ We note that as the ethanol that was used to rinse the films after the acid etching evaporated, lateral capillary forces caused the nanowires to bend and cluster, forming bundles with a nearest neighbor center‐to‐center spacing of ≈400 nm at the tips.

**Figure 2:**
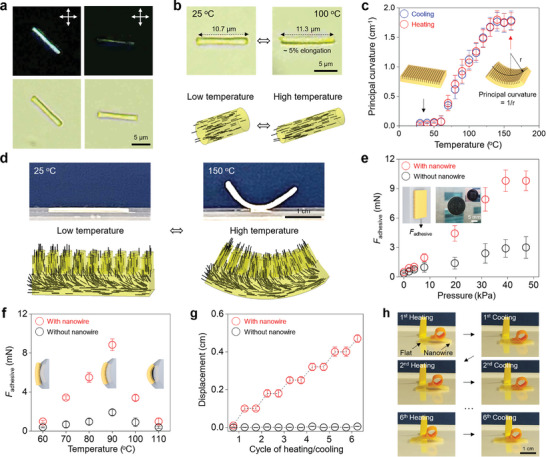
Temperature‐induced adhesion and shape deformation of LCE nanowire films. a) Polarized light and bright field micrographs of individual LCE nanowires. The crossed double‐headed arrows indicate the orientation of the crossed polarizers. b) Polarized light micrographs and computer simulation showing the deformation of individual LCE nanowires upon an LC–isotropic phase transition. The black lines in the computer simulations represent the orientation of the LC moieties. c) The principal curvature of LCE nanowire films as a function of temperature. The insets show schematic illustrations of the LCE nanowire films at low and high temperatures. d) Computational simulation and photographs of the deformation of LCE nanowire films upon an LC–isotropic phase transition. The black lines in the computer simulations represent the orientation of the LC moieties. e) Adhesive forces of LCE films with and without nanowires on vertical glass surfaces as a function of applied pressure, which was held for 3 s before measuring the adhesive force. The inset shows a schematic of the experiment and a photo of a 10 mg LCE nanowire film (5 mm × 5 mm) holding an ≈2.3 g U.S. dime against a glass window after applying a 40 kPa pressure for 3 s. f) Adhesive forces of LCE films with and without nanowires on a curved glass surface with a curvature of ≈0.9 cm^−1^ as a function of the temperature. A 40 kPa pressure was applied for 3 s before measuring the adhesive force. The insets show schematic illustrations of the curvature matching between the LCE nanowire films and curved glass surfaces. g,h) Plot and corresponding photographs showing the displacement of an LCE film with the nanowires removed from the left end, caused by cycling the temperature to induce reversible curling. No displacement was observed when all of the nanowires were removed. Error bars represent standard deviations from three independent measurements for each data point.

The internal alignment of the LC moieties within the nanowire structures was characterized using polarized light microscopy and stimuli‐responsive deformations. As shown in the representative polarized light micrographs of an individual nanowire (**Figure** **2**a), the nanowires exhibited a bright‐dark optical transition when rotated between crossed polarizers, with maximum brightness at 45° with respect to the crossed polarizers. For increased visibility, the nanowire was imaged at a small angle away from the alignment of the crossed polarizers in the right set of images. This observation indicates that the LC moieties are aligned along one of the nanowire's axes. Upon further analysis, when heated, the individual nanowire elongates along its major axis, with deformation onset happening at ≈65 °C, as evidenced by the bright field micrographs and computational simulation of an individual nanowire shown in Figure 2b. The elongation of the long axis, accompanied by a shrinkage in the short axis, points toward a polymer chain alignment in the direction of the short axis, a characteristic of an oblate LCE. This is consistent with past studies, where side‐chain end‐on LCEs typically exhibit an oblate conformation (where the polymer chains are aligned perpendicular to the alignment of the LCs).^[^ ^24^ ^]^ This oblate LCE leads to an anisotropic shape elongation along the LC alignment, where the radius of gyration perpendicular to the LC alignment is larger than the radius of gyration parallel.

In the next set of experiments, we investigated the thermal‐induced shape changes in the entire LCE nanowire film. When heated above the onset temperature, the films curl toward the nanowire‐covered side, as demonstrated in the photographs and schematics in Figure 2d. As shown in Figure 2c, this curling deformation begins at the deformation onset temperature and continues until ≈130 °C, where a high‐temperature plateau is reached. As shown in the DSC results in Figure S3 (Supporting Information), the films exhibit a broad transition that matches the observed thermal‐induced shape deformation. The broad transition may be attributed to variations in polymerization rates and alignments throughout the film (see detailed discussion in the Supporting Information). Our X‐ray scattering analysis reveals that, upon polymerization, this mixture of end‐on LC monomer and crosslinker transitions from a nematic phase to a smectic A phase (Figure S4, Supporting Information), consistent with prior studies.^[^ ^24^ ^]^ The curling deformation is attributed to differing LC alignments at the surfaces of the LCE film: on the nanowire side of the film, the majority of the LC molecules are expected to have a perpendicular orientation relative to the surface of the film caused by the alignment within the nanovoids in the AAO, whereas the LCs adopt a uniform parallel alignment on the side in contact with the polyimide‐coated glass slide. The deformation of the individual LCE nanowires and LCE nanowire films is shown in computational simulations found in Supporting Movie 1. The maximum principal curvature of the film is found to depend on the thickness of the LCE film rather than the nanowire length, with thicker films curling less than thinner ones, as shown in Figure S5 (Supporting Information). Furthermore, we observed that the film still bends toward the nanowire surface when the nanowires are removed, indicating that the nanowires themselves are not inducing the curling deformation, though their existence during polymerization does provide for the alignment within the film. In addition, LCE films made without the AAO template but with the same surface alignments showed curling deformations in the direction of what would be the nanowire side (see Figure S6, Supporting Information).

Given the impact of nanowires on surface adhesion and friction reported in previous studies, we explored their effect on the surface adhesion of LCE films. We placed the nanowire‐covered film onto a vertical glass slide with the nanowires in contact with the slide, applying a normal pressure for 3 s to promote adhesion. We then measured the adhesive shear force—the maximum force parallel to the film/glass interface at which the film slid off the slide. Figure 2e shows that the presence of nanowires significantly enhanced the adhesive shear force of the LCE film, increasing it fivefold compared to a film that had the nanowire structures removed. Additionally, increasing the normal pressure allowed the film to support greater weight until a maximum adhesive shear force was reached. As shown in the inset of Figure 2e, a 10 mg square of LCE nanowire film (≈5 mm by 5 mm) could hold a 2.3‐g American dime, 230 times the mass of the LCE film. Furthermore, we used theoretical models to investigate the impact of the diameter of the nanowires on the increased adhesion and found that nanowires with larger diameters are expected to exhibit enhanced adhesion to both solid and liquid surfaces (see Figure S7, Supporting Information).

In addition to the enhanced adhesion, the film's curling ability synergistically combines with the nanowire structures, enabling it to conform to convex surfaces by increasing the contact area, as illustrated in Figure 2f. When adhered to a convex surface, there exists an optimized temperature at which the film's curvature best matches the surface curvature, maximizing adhesion. Deviating from this optimal temperature, either by heating too much or too little, disrupts this curvature match, reducing the adhesion strength. Alternatively, the curling deformation can be used in conjunction with the enhanced adhesion from the nanowire structures to cause the films to mimic inchworm‐like crawling. As shown in Figure 2g and photographed in Figure 2h, a patterned nanowire‐coated LCE film exhibited curling and flattening cycles when subjected to temperature cycling above and below the deformation onset temperature, enabling it to crawl along a flat glass slide. Specifically, the frictional asymmetry introduced by removing nanowires from one side creates an imbalance that governs the film's curling behavior. Upon heating, the film undergoes its typical curling motion, but this process detaches the nanowire side from the glass slide while the flat side adheres more effectively. This differential adhesion anchors the flat side, allowing the curling motion to move the nanowire side. During cooling, the nanowire side provides increased sliding friction, anchoring itself to the glass slide and pushing the flat side forward. By cycling the temperature, this alternating anchoring and motion enable the film to exhibit a crawling motion. This curling action can also lift small additional weights comparable to the film's own weight.

**Figure 3:**
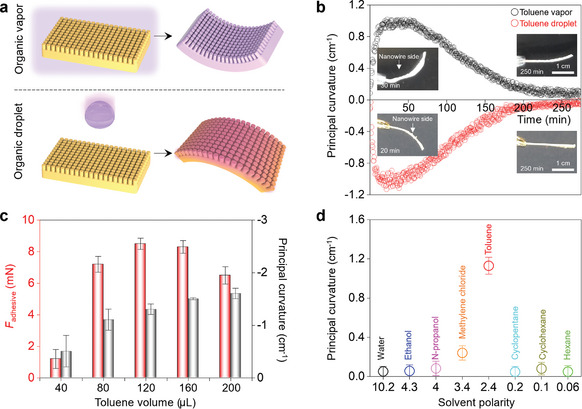
Chemical‐induced shape deformation of LCE films with nanowire structures. a) Schematic of the shape deformations of LCE nanowire films under the presence of an organic solvent vapor condition and after the application of an organic solvent droplet condition. b) Principal curvatures of LCE nanowire films as a function of time immersed in toluene vapor (24 ppm) and after the application of a toluene droplet (120 µL). The toluene vapor was removed at 20 min. c) Maximum adhesive forces and principal curvatures of LCE nanowire films as a function of applied toluene droplet volume. A 40 kPa pressure was applied for 3 s before measuring the adhesive force. d) Principal curvatures of LCE nanowire films as a function of the solvent polarity of 120 µL organic solvent droplets. Error bars represent standard deviations from three independent measurements for each data point.

These results demonstrate that the stimuli‐responsive deformations of the LCE nanowire films can be precisely activated and programmed through temperature changes. In addition to temperature control, we explored the intrinsic response of our LCE nanowire films to other types of stimuli, such as solvent‐induced swelling. As illustrated in **Figure** **3**a, when the LCE nanowire films were exposed to the vapor of an appropriate chemical, the vapor swells the film, weakening the interactions between the LC moieties and disrupting the alignment of the LC moieties, similar to the process of the thermal‐induced LC–isotropic phase transition.^[^ ^25^ ^]^ This reduction in LC alignment causes the film to deform and curl in the same direction as observed under heating, that is, curling toward the nanowire‐covered side. However, when the film was immersed in bulk toluene or when a toluene droplet was placed on the film surface, the film curled away from the nanowire‐covered side, exhibiting an opposite response—this behavior has not been achieved with this system during thermally‐induced shape deformation. This response is attributed to capillary forces drawing the toluene toward the nanowire side, resulting in an unequal swelling of the film. The swelling causes the nanowire side to expand, while the other side experiences less expansion, leading to the observed curling away from the nanowire side. To further investigate the role of nanowires, the solvent‐induced deformation of films after the nanowires were removed and films without nanowires but with the same hypothesized alignment were investigated. As shown in Figure S8 (Supporting Information), both films deformed toward the side corresponding to the nanowire surface. This deformation aligns with the behavior observed in films exposed to solvent vapors or elevated temperatures but contrasts with the response of LCE nanowire films, which curl in the opposite direction when exposed to a solvent droplet. This result further supports the hypothesis that the nanowires play an important role in this unique reversed deformation. Polarized light micrographs of the film before and after swelling can be seen in Figure S9 (Supporting Information). The disappearance of the bright‐to‐dark transition upon rotation between crossed polarizers after immersion in toluene points toward the swelling of the film and disruption of the LC moieties’ alignment. Notably, in both solvent types, the film recovers to its original shape once the toluene evaporates, as shown in Figure 3b.

In contrast to the curling induced by temperature, which primarily matches convex shapes by curling toward the nanowire‐covered side of the LCE film, the deformation caused by a toluene droplet can be synergistically combined with the enhanced adhesion of the nanowire structures to conform to concave substrates, as depicted in Figure 3c. When the curvature of the LCE film optimally matches that of the substrate, the adhesive force is maximized. Our investigations into other solvents revealed that a specific level of polarity is necessary to induce deformations in the film, as demonstrated in Figure 3d. The solvent polarity selectivity arises from conventional solvent–polymer interactions, where solvents with polarities similar to the polymer promote favorable interactions and sufficient solubility.^[^ ^25^ ^]^ LC polymers exhibit dual characteristics, combining polar functional groups (e.g., ester groups) with nonpolar aromatic rings. This unique composition makes solvents of intermediate polarity, such as toluene, particularly effective for swelling the LC polymers. In contrast, highly polar solvents (e.g., ethanol) and highly nonpolar solvents (e.g., hexanes) are inefficient for swelling. The enhanced solubility achieved with intermediate‐polarity solvents facilitates greater solvent vapor uptake, thereby amplifying the deformation response. Overall, the films exhibit responsiveness to both temperature and chemical stimuli, with the friction amplification provided by the nanowire structures synergizing with the curling deformations to maximize adhesion to curved surfaces.

**Figure 4:**
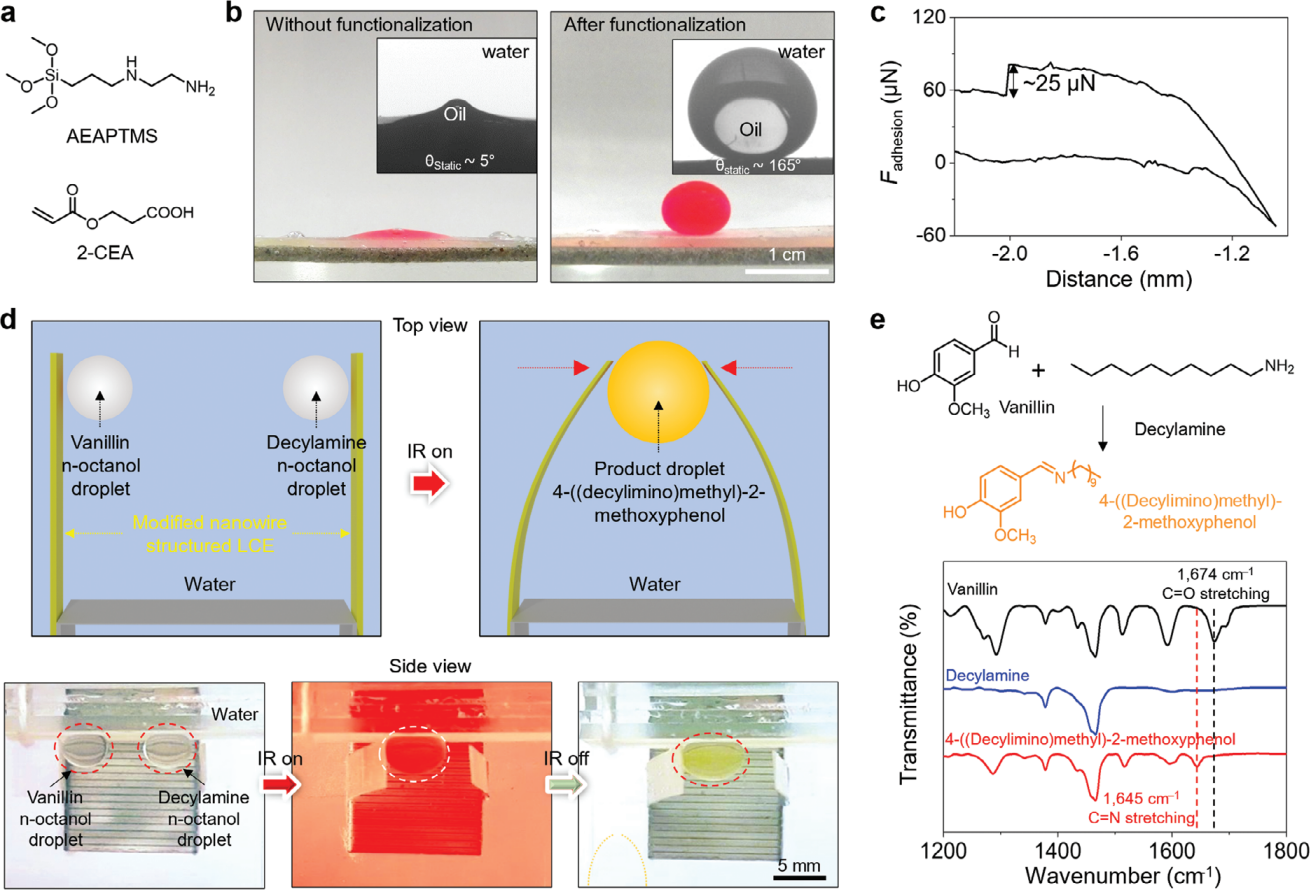
Underwater superoleophobicity of LCE nanowire films induced by chemical modification. a) Molecular structures of AEAPTMS and 2‐CEA. b) Photographs and contact angle goniometer images showing the underwater oil wettability of LCE nanowire films before and after surface modification. c) Underwater adhesive force of a 5 µL dichloromethane droplet on chemically modified LCE nanowire films. d) Chemical reaction between colorless vanillin and decylamine producing yellow 4‐((decylimino)methyl)‐2‐methoxyphenol, driven by an LCE nanowire film‐based droplet mixer. Two 10 µL colorless n‐octanol oil droplets containing vanillin (left) and decylamine (right) were placed on an underwater superoleophobic substrate. IR light‐induced actuation of the LCE nanowire films (fixed at one end) mixed the droplets. e) ATR‐FTIR spectra showing the disappearance of the C═O peak at 1674 cm^−1^ and the appearance of the C═N peak at 1645 cm^−1^, confirming the reaction between the amine and aldehyde.

In the final set of experiments, we investigated the wettability of the LCE nanowire films in both air and underwater conditions (Figure S10a, Supporting Information). The LCE nanowire films exhibit intrinsic hydrophobicity with a water contact angle of ≈148°, as shown in the contact angle goniometer image in Figure S10 (Supporting Information). Without further surface modification, Figure S11a (Supporting Information) demonstrates that the nanowires enhance the adhesion of water to the LCE surface, allowing micrometer‐sized water droplets to be lifted. This adhesive effect of the nanowires is further illustrated graphically in Figure S11b (Supporting Information). However, when the films were submerged underwater with an oil droplet on top, specifically dichloroethane, the droplet spread across the surface (**Figure** **4**b), likely due to capillary forces similar to those observed during the solvent‐induced shape deformation, as shown in Figure 3c.

To provide the LCE nanowire films with underwater antifouling properties, similar to structured surfaces like filefish skin, we applied a simple surface modification to the LCE film using 3‐(2‐aminoethylamino)propyltrimethoxysilane (AEAPTMS) and 2‐carboxyethylacrylate (2‐CEA), and their molecular structures are provided in Figure 4a. This chemical modification was confirmed through attenuated total reflectance‐Fourier transform infrared (ATR‐FTIR) spectroscopy, as shown in Figure S12 (Supporting Information). Following this modification, the LCE nanowire films exhibited underwater superoleophobicity to various oil droplets, as evidenced by Figure 4b and Figure S13 (Supporting Information). The adhesion force of a 5‐µL dichloroethane droplet was determined to be ≈25 µN, measured following its compression to a modified LCE nanowire film with a preload force of 50 µN and subsequent detachment, as shown in Figure 4c. This superoleophobicity remains stable under harsh conditions over a period of 30 days, including extreme pH values, artificial seawater, and surfactants, as shown in Figure S13b (Supporting Information). Furthermore, the nanowire structures enhanced the underwater superoleophobicity of the modified LCE films, indicated by a reduction in contact angle when the nanowires were removed (Figure S11b, Supporting Information).

By integrating this underwater superoleophobicity with the stimuli‐responsive deformation of the LCE films, we developed an underwater droplet‐mixing system using two LCE nanowire films. As illustrated in Figure 4d, droplets placed at the ends of two LCE nanowire films submerged underwater were mixed by heating with a 150 W, broad wavelength infrared bulb, as further described in the Supporting Information, causing the curling of the films to facilitate a chemical reaction. Specifically, we demonstrated a colorimetric reaction between vanillin (colorless) and decylamine (colorless) to yield 4‐((decylimino)methyl)‐2‐methoxyphenol (yellow), as shown in Figure 4d. The reaction was verified through ATR‐FTIR, UV–vis, and ^1^H NMR spectroscopy (Figures S14 and S15, Supporting Information), which confirmed the disappearance of the C═O bond in vanillin and the emergence of the C═N bond in the final product (Figure 4e). Notably, the curling of the LCE nanowire films was stable across temperature changes (Figure S16a, Supporting Information). Additionally, the chemical modification preserved the reversible deformation characteristics of the LCE films, as demonstrated in Figure S16b (Supporting Information).

Moreover, the inherently hydrophobic nature of the LCE nanowire films in air exhibited an extreme repellence to water droplets when submerged in oil. Leveraging their temperature‐responsive curling behavior, we designed an underoil water droplet‐mixing robot, where two LCE nanowire films facilitate a chemical reaction between iron(III) chloride (colorless) and potassium thiocyanate (colorless) to achieve a blood red colored product, as illustrated in Figure S17 (Supporting Information). These results highlight the versatility of the chemical modification in tuning the super wettability while maintaining the shape deformability of the LCE nanowire films.

## 3 Conclusion

In this work, we reported the design and synthesis of LCE nanowire films that exhibit distinct stimuli‐responsive deformations when subjected to thermal and solvent stimuli. The nanowire structures were shown to increase the adhesion of the films to surfaces, mimicking biological systems. Additionally, the AAO template used to create the nanowires on the surface of the LCE films created a uniform alignment within the LC molecules, confirmed through polarized light microscopy and stimuli‐responsive shape deformations, which also aligned the LC moieties within the film perpendicular to the surface. Because of this alignment and the parallel alignment on the other side of the film, the LCE films curl toward the nanowire covered surface upon heating or exposure to solvent vapors, with the ability to revert to their original shape upon removal of the stimulus. However, exposure to a solvent droplet causes the films to curl in the reverse direction. The combined effect of curling and increased adhesion allows the films to conform to both convex and concave surfaces. Additionally, the nanowire structures enhanced the film's antifouling properties in water and oil. A simple chemical modification introduced underwater superoleophobicity, expanding potential applications in harsh environments. The development of an underwater droplet‐mixing robot further demonstrates the practical utility of these materials in facilitating chemical reactions in aqueous environments.

Future efforts will explore the systematic optimization of the nanowire fabrication process to tailor the structural properties of LCE films for specific applications. Additionally, investigating the interplay between nanowire dimension, LC alignment, and environmental stimuli can yield deeper insights into the design of advanced materials with programmable responses. The temperature‐induced adhesion, shape deformation, and inchworm‐like crawling motion of LCE nanowire films can be exploited for various functionalities such as artificial muscles, pumping, locomotion mechanisms, soft grippers, and applications in biomedical fields.^[^ ^26^ ^]^ These lightweight, responsive films are particularly suitable for delicate tasks, such as handling fragile objects or biomedical samples with precision. Another demonstrated promising application is the use of antifouling LCE nanowire films, with or without modification, for automated liquid droplet manipulation without incurring any mass loss. The inherent extreme liquid repellence ensures no mass loss caused by adhesion to a solid surface while manipulating the liquid droplets with the stimuli‐responsive films. This enables precise liquid control in small volumes without having adhesion‐based mass loss, enhancing experimentation efficiency and minimizing human error. Such capabilities can be extended to lab‐on‐a‐chip systems and microfluidic platforms,^[^ ^27^ ^]^ enabling microreactions and miniaturized biological laboratory processes. By leveraging the unique characteristics of LCEs and nanostructures, we aim to further explore their capabilities in dynamic environments, paving the way for innovative solutions in materials science and engineering.

## Conflict of Interest

The authors declare no conflict of interest.

## Supporting information



Supporting Information

Supplemental Movie 1

## Data Availability

The data that support the findings of this study are available in the supplementary material of this article.
