# Supplementary material for: Synergistic Adhesion and Shape Deformation in Nanowire‐Structured Liquid Crystal Elastomers
Source: Adv Mater. 2025 Jan 19;37(9):2414695. doi: 10.1002/adma.202414695 (PMC11881676; doi:10.1002/adma.202414695)
Supplement: Supplementary file 1 — Supporting Information [file ADMA-37-2414695-s001.docx]

Supporting Information

**Synergistic Adhesion and Shape Deformation in Nanowire-Structured Liquid Crystal Elastomers**

Robert L. Dupont, Yang Xu, Angana Borbora, Xinyu Wang, Fatemeh Azadi, Kaden Havener, Broderick Lewis, Weichen Deng, Benjamin W. Tan, Shucong Li, Rui Zhang, Yuxing Yao, Uttam Manna, Xiaoguang Wang

**Methods and Materials**

**Materials**

The following liquid crystal (LC) monomers were purchased from Synthon Chemicals Ltd.: LC monomer 4-(6-acryloxy-hex-1-yl-oxy)phenyl 4-(hexyloxy)benzoate and LC crosslinker 1,4-bis-[4-(3-acryloyloxypropyloxy)benzoyloxy]-2-methylbenzene (RM257). The following chemicals were purchased from Sigma-Aldrich: 10 cSt silicone oil, photoinitiator 2,2-dimethoxy-2-phenylacetophenone (DMPAP), dimethyloctadecyl [3-(trimethoxysilyl)propyl] ammonium chloride silane (DMOAP), poly(pyromellitic dianhydride-co-4,4’-oxydianiline) amic acid, 1-methyl-2-pyrrolidinone, 3-(2-aminoethylamino)propyltrimethoxysilane (AEAPTMS), 2-carboxyethylacrylate (2-CEA), dichloromethane, iron(III) chloride (FeCl_3_), potassium thiocyanate (KSCN), hexane, n-heptane, carbon tetrachloride, chloroform, n-octanol, toluene, hydrochloric acid (HCl), sodium dodecyl sulfate (SDS), dodecyltrimethylammonium bromide (DTAB), branched polyethyleneimine (BPEI; molecular weight 25,000 Da), tripentaerythritol pentaacrylate (5Acl; molecular weight 524.21 Da), 3-(dimethylamino)-1-propylamine, octadecyl amine, pentanol, vanillin and decyl amine. Ethanol was purchased from Tedia Company, USA. Anodic aluminum oxide (AAO) was purchased from Shanghai Shangmu Technology Co. Ltd, China. Plain microscope slides (25 mm × 75 mm × 1 mm) were purchased from Fisher Scientific. Unless stated otherwise, purchased chemicals and materials were used as received without further modification or purification.

**Synthesis of liquid crystal elastomer (LCE) nanowire films**

First, we prepared a polyimide-coated glass slide by spin coating a poly(pyromellitic dianhydride-co-4,4’-oxydianiline) amic acid in 1-methyl-2-pyrrolidinone solution on a glass slide at 2,000 rpm. This glass slide was then heated at 350℃ for 2 hours before rubbing the coated side with velvet to create the planarly aligning polyimide coated glass slide. We then placed an AAO template on a separate, uncoated glass slide along with two spacers. Fig. S1 in the Supporting Information shows a representative SEM image of the top and side of the templates. The templates used created nanowires that were 10 µm long and 400 nm in diameter. This was preheated at 65℃ while the following steps were performed. A mixture of 90 wt% end-on and 10 wt% RM257 was mixed together and heated at 80℃ to melt. Mixing was done through vortex. Then, an additional 1 wt% of photoinitiator, DMPAP, was added to the mixture before mixing briefly with vortex. This mixture was then pipetted onto the AAO template and was allowed to sit for 10 minutes so that the mixture could fill the voids in the template. The polyimide-coated glass slide was placed on top, and the optical cell was clipped together where the spacers were. This was then polymerized with 365 nm UV for 15 minutes. Once done, the glass slides were separated and the back of the AAO template was scratched with a pair of tweezers. The AAO template, LCE film, and glass slide stuck to the LCE film were then placed in 12 M HCl until the AAO template was fully etched away, usually around 4 days. Finally, the film was taken from the HCl, washed with water, and dried for further testing.

**Characterization of morphology of nanowires on LCE films**

The scanning electron microscope (SEM) images of nanowires on LCE films were obtained using a JEOL JSM-7800F Prime SEM instrument. Before testing, all samples needed to be coated with a conductive gold layer (~ 10 nm) prior to analysis with the SEM.

**Theoretical computation of LCE nanowire film deformation**

Simulations were performed using Abaqus/CAE 2019 with C3D8 elements (3D, eight-node). The model geometry matches experimental dimensions, and a director field was introduced to represent the nematic director of the LC, satisfying the film’s anchoring condition. Direction 1, aligned with the nematic director, was assigned to model anisotropic expansion, where the expansion coefficient along this direction differs from the others. A uniform strain field was applied to simulate stimuli-responsive deformation.^[1]^

**Thermal-induced shape deformation of LCE nanowire films**

A strip of film approximately 10 mm long and 2 mm wide was cut with a scalpel from the larger film. This strip was then put into a beaker of silicon oil on a hotplate. A thermometer was placed into the silicon oil to accurately measure the temperature. The temperature of the hotplate was then changed and allowed to stabilize before a picture of the film was taken. The principal curvature of the curling film was then measured using a homemade MATLAB program.

**Solvent-induced shape deformation of LCE nanowire films**

A strip was cut from the full film as before. For the solvent vapor deformation testing, the film was gently grasped by the arms of a binder clip which was then placed on its side. This was done to hold the film strip above the surface of the table. A pool of solvent was placed below the film making sure that the film did not touch the solvent. A crystallizing dish was then placed over top of the film and pool of solvent to trap the solvent vapor. A video of the film was taken and was fed into the same MATLAB program to be analyzed. The program was designed to take frames from the video every 10 seconds to speed up processing and analysis.

For the solvent droplet deformation testing, the film was again gently grasped by the arms of a binder clip which was then placed on its side to hold the film off the table. Then, a droplet of solvent was carefully placed on the edge of the film. A video of the film was taken like before and was analyzed in MATLAB.

**Temperature-mediated adhesion of LCE nanowire films**

A strip was cut in the same ways as before. A small hook was attached to the flat side of the film so that the testing weights could be hung from the sample. The sample was then placed against the desired substrate with the nanowire side touching the substrate. Water was then put into a small container which was placed on the film and used to control the force pressing the sample to the substrate. The substrate was then lifted up and the desired weight was hung to the sample. This setup was placed into an oven to control the temperature. More weight was added until the film slipped off of the substrate. Images were taken and the force of the weight hung to the film was compared to the force used to adhere the film to the substrate.

**Solvent-mediated adhesion of LCE nanowire films**

The same basic setup was used for the solvent controlled adhesion testing as was used for the temperature-controlled adhesion testing. Small droplets of solvent were then placed under the film similar to the method used to test the solvent vapor-based deformation of the films. More weight was added until the film slipped off of the substrate. Images were taken and the force of the weight hung to the film was compared to the force used to adhere the film to the substrate.

**Surface chemical modification of LCE nanowire films**

Underwater superoleophobicity in LCE nanowire films was achieved through a 1,4-conjugate addition reaction between the amine group of AEAPTMS and the acrylate groups of 2-CEA, applied using a dip-coating method. Strips of LCE nanowire films (approximately 10 mm long and 2 mm wide) were immersed in an ethanol solution containing AEAPTMS (2.22% v/v) and 2-CEA (1.20% v/v) for 6 hours, followed by washing in ethanol and air drying. The success of this chemical functionalization was verified via attenuated total reflection Fourier transform infrared (ATR-FTIR) spectroscopy, recorded with a PerkinElmer UTAR Two instrument at ambient conditions.

**Contact angle measurements of LCE nanowire films**

Water contact angles on the LCE nanowire films, both before and after surface modification, were measured using a KRUSS 25E Drop Shape Analyzer. The measurements were conducted using 5 µL water droplets placed at three distinct locations on each LCE film. Similarly, oil contact angles were measured using 5 µL oil droplets at three different locations on the LCE films.

**Measurement of adhesion forces on LCE nanowire films**

The underwater adhesion force of oil droplets on the modified LCE nanowire film was evaluated using a microelectromechanical balance system (Kruss force tensiometer, Germany). A 5 μL dichloromethane droplet, suspended on a metal holder, was brought into contact with the film under a controlled preload force (50 µN). Following contact, the droplet was retracted from the surface, and the force changes during this process were measured by a highly sensitive microbalance.

**Fabrication of LCE nanowire film-based droplet mixers**

To design a water droplet-mixing robot, two LCE nanowire films were fixed parallel, 20 mm apart, with the nanowire sides facing inward. One end of each LCE was glued to a support. Two colorless aqueous reactant droplets (10 μL) containing 0.1 M FeCl_3_ and 0.1 M KSCN were placed on the free ends of the LCE films on a superhydrophobic surface, as shown in Figure S17a. The superhydrophobic surface was created by spraying a pentanol solution of 10 mL of 5Acl (132.5 mg/mL) and 3 mL of BPEI (50 mg/mL) onto glass substrates (75 mm × 25 mm) from a 15 cm distance. After solvent evaporation, the formed porous polymeric coating was post-functionalized with octadecyl amine (2.5 mg/mL in ethanol) for 12 hours via 1,4-conjugate addition to achieve superhydrophobicity.^[2]^ Infrared light exposure gradually heated the oil and films, inducing curling of the LCE films toward each other, mixing the reactants, and producing a blood-red solution, as shown in Figure S17b.

For underwater oil droplet mixing, a similar setup used chemically modified LCE nanowire films with underwater superoleophobicity. Submerged in water, two colorless reactant droplets of vanillin (50 mg/mL) and decylamine (65.5 μL/mL) in n-octanol were placed near the ends of the LCE films on an underwater superoleophobic surface using a J-shaped needle syringe (Figure 4d). The underwater superoleophobic surface was prepared by mixing ethanol solutions of 5Acl (132.5 mg/mL) and BPEI (50 mg/mL) in a 1:10 ratio, forming reactive nanocomplexes in 5 minutes. These were applied to glass substrates using a layer-by-layer process, alternating 10-second immersions in BPEI and nanocomplex solutions with ethanol rinsing between steps.^[3]^ The coating was then treated with 3-(dimethylamino)-1-propylamine (2.5% v/v in ethanol) for 12 hours to achieve underwater superoleophobicity.

Infrared light was emitted from a 150 W IR bulb with a broad wavelength spectrum, which peaks at 1000 nm, and was applied for 15 minutes from a distance of 10 cm to raise the temperature from 30ºC to 80ºC of a water bath of 45 mL. Upon IR irradiation, the gradual heating caused the films to curl toward each other, mixing the reactant droplets to form a yellow product (Figure 4d). The final product, 4-((decylimino)methyl)-2-methoxyphenol, was confirmed using a Thermo Scientific Multiskan GO UV-visible spectrophotometer and a Varian Mercury Plus 400 MHz nuclear magnetic resonance (NMR) spectrometer. ^1^H NMR (400 MHz, CDCl_3_) of 4-((decylimino)methyl)-2-methoxyphenol: δ (ppm) 8.15 (s, 1H), 7.44 (s, 1H), 7.08 (d, 1H), 6.91 (d, 1H), 3.84 (s, 3H), 3.59 (t, 2H), 1.7 (m, 2H), 1.27 (m, 14H), 0.89 (t, 3H).

**Thermal characterization of LCE**

The phase transition temperatures of the LC monomer/crosslinker mixture and their corresponding LCE were determined using differential scanning calorimetry (DSC) on a TA DSC Q100 instrument. The heating and cooling rates for the DSC measurements were set to 2°C/minute.

**X-ray scattering analysis of LCE**

The phase behavior of the LC moieties within the LCE was investigated using wide-angle/small-angle X-ray scattering (WAXS/SAXS). Measurements were performed on a Xenocs Xeuss 2.0 laboratory beamline system with an X-ray wavelength of 1.54 Å. Samples were placed in a large vacuum chamber to reduce air scattering. Diffraction patterns of the LC polymers were recorded with a Pilatus 1M detector (Dectris Inc.) over an exposure time of 1.5 hours and processed using the Nika software package and WAXSTools in Wavemetrics Igor. The detector was positioned 15 cm from the sample to capture detailed structural information.

S**tatistical analysis**

All averages were numerical averages, and all confidence intervals were calculated from samples sizes (n) equal to 3.

**Results**

**Differential scanning calorimetry and X-ray scattering characterization of LCE nanowire films**

DSC was used to investigate the phase transition temperatures of the LCE film and LC mixture. The DSC results can be seen in Figure S3. The T_N–I_ of the mixture was found to be about 65°C. A broad transition was observed in the LCE film ranging from about 65°C to 130°C, which is in good alignment with the thermal-induced deformations observed in the films (Figure 2c). Our WAXS/SAXS analysis revealed that, upon polymerization, this mixture of end-on LC monomer and crosslinker transitions from a nematic phase to a smectic A (SmA) phase (Figure S4), consistent with prior studies.^[4]^

The broad transition may be attributed to variations in the polymerization rates induced by the nanoscale structure of the AAO template, which results in compositional heterogeneity within the LCE nanowires and into the LCE film. This heterogeneity may arise from non-uniform light intensity during photopolymerization, diffusion limitations of reactants, and surface effects affecting local polymerization kinetics. Additionally, variations in heat dissipation during the exothermic reaction could further contribute to these differences. Finally, variations in the UV power through the film likely affects the polymerization kinetics which would cause additional heterogeneity.

**Shape deformation of flat and nanowire-removed LCE films**

To further investigate the role of nanowires in the curling deformation of LCE films, we removed the nanowires from the surface of a film and subjected it to heating. As shown in Figure S5, the removal of nanowires had minimal to no effect on the maximum curling deformation of the LCE film. Images of the curling deformation for the nanowire-removed LCE films are provided in Figure S6a, where the side that previously supported the nanowire structures is oriented upwards. The deformation direction remains consistent with that of films that still have nanowires.

To validate our hypothesis regarding the oblate alignment of the LCE, we also examined thermally induced deformation in LCE films fabricated without nanowires but with the same proposed alignment. Specifically, one side of the film was anchored to a polyimide-coated slide, while the other side used a DMOAP-coated slide instead of an AAO template, to create a homeotropic alignment. As shown in Figure S6b, the flat LCE film curls in the absence of nanowires and does so in the direction of the homeotropically aligned side that would typically support the nanowires.

These results support our hypothesis that the AAO template induces planar anchoring of the LC moieties within the nanovoids, which translates into perpendicular alignment of the LCs at the surface. This alignment causes the polymer chains on the AAO side to align parallel to the surface, while the chains on the polyimide side align perpendicular to the surface. The resulting anisotropic alignment drives the observed curling deformation towards the AAO side.

In addition to the thermally activated experiments, we investigated the response of these films to a 40 µL droplet of toluene. As shown in Figure S8, when toluene was applied to flat and nanowire-removed LCE films, the films curled toward the side promoting perpendicular LC ordering, such as the AAO template or the DMOAP-functionalized glass slide. This behavior contrasts with the nanowire films, which curl away from the nanowire side under similar conditions. Notably, this curling direction is consistent with the film’s response to both toluene vapor and temperature stimuli. These observations support our hypothesis that the unequal swelling of the LCE nanowire film, driven by higher toluene concentrations due to capillary forces from the nanowire structures, is responsible for the observed behavior. Polarized optical micrographs of the swollen film, provided in Figure S9, reveal the disappearance of the characteristic bright-to-dark transition. This change indicates film swelling and a loss of LC orientation due to solvent-induced disruption.

**Adhesion to solid surfaces**

For adhesion to solid surfaces, we use the Johnson–Kendall–Roberts (JKR) model to describe the adhesion force between an individual LCE nanowire and a solid surface:^[5]^

$F_{nanowire-solid}= \frac{3}{2}\pi R_{nanowire}W_{nanowire-solid}$ (1)

where *F*_nanowire–solid_ is the adhesion force, *R*_nanowire_ is the radius of the LCE nanowire, and *W*_nanowire–solid_ is the work of adhesion between the LCE and the solid surface. Considering the density of LCE nanowires, the total adhesion force between the LCE nanowire film and the solid surface (*F*_nanowire–solid,total_) can be written as:

$F_{nanowire-solid,total}= n_{nanowire}F_{nanowire-solid}$ (2)

where *n*_nanowire_ is the number density of LCE nanowires per unit area.

Based on the above JKR model, for a given number density of LCE nanowires, the adhesion force to the solid surface increases proportionally with the nanowire diameter.

**Adhesion to liquids**

Since the LCE material used in this study is hydrophobic, we use the theory of liquid droplet adhesion on superhydrophobic materials to elucidate the behavior of droplets on LCE nanowires. The adhesion of the droplets on the LCE nanowire film (*F*_nanowire–liquid,total_) is primarily determined by the contact angle hysteresis of liquid droplets on the film:^[6]^

$F_{nanowire-solid,total}= \gamma_{liquid}L_{nanowire-liquid}(\cos\theta_{r}-\sin\theta_{a})$ (3)

where *γ*_liquid_ is the surface tension of the liquid, *L*_nanowire–liquid_ is the total contact line length between the LCE nanowire film and the liquid droplet on the film, and *θ*_r_ and *θ*_a_ are the receding and advancing contact angles, respectively. The difference between *θ*_a_ and *θ*_r_ is defined as the contact angle hysteresis. *L*_nanowire–liquid_ can be estimated as the sum of the circumference of every LCE nanowire under the droplet contact line, as:

$L_{nanowire-liquid}= \frac{4\pi^{2}R_{liquid}R_{nanowire}}{p}$ (4)

where *R*_liquid_ is the radius of the liquid droplet on the LCE nanowire film and *p* is the center-to-center spacing between the bottom of the LCE nanowires.

For a given number density of LCE nanowires, as the nanowire diameter increases, *L*_nanowire–liquid_ increases proportionally, leading to a higher adhesion force. Therefore, we estimate that LCE nanowire films with larger diameter nanowires will exhibit stronger liquid adhesion compared to those with smaller diameter nanowires, assuming all other factors remain constant.

To validate our hypothesis, we conducted experiments using LCE nanowire films with identical number density (pitch is 450 nm) and length but varying diameters (400 nm and 200 nm). The results demonstrate that:

1. Solid surface adhesion: LCE films with 400 nm-diameter nanowires exhibit approximately two times the total adhesion force compared to those with 200 nm-diameter nanowires.
2. Liquid adhesion: LCE films with 400 nm-diameter nanowires show approximately twice the total liquid adhesion force relative to those with 200 nm-diameter nanowires.

These findings can be found in Figure S7 and align with our theoretical predictions based on the Johnson–Kendall–Roberts model for solid adhesion and the contact line theory for liquid adhesion.


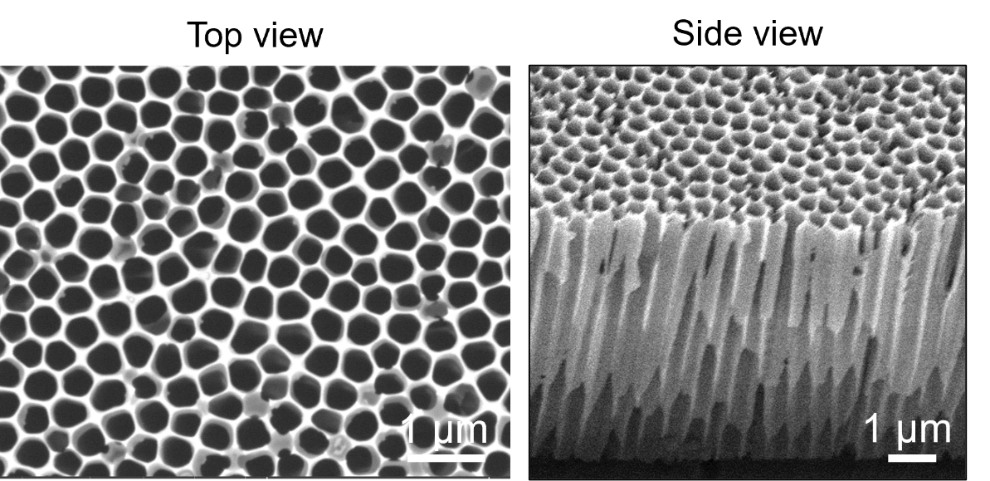


**Figure S1. SEM images (top and side views) of anodized aluminum oxide (AAO) templates.** The diameter of the pores is 400 nm, and the depth is 10 μm. The templates were not coated for the imaging.


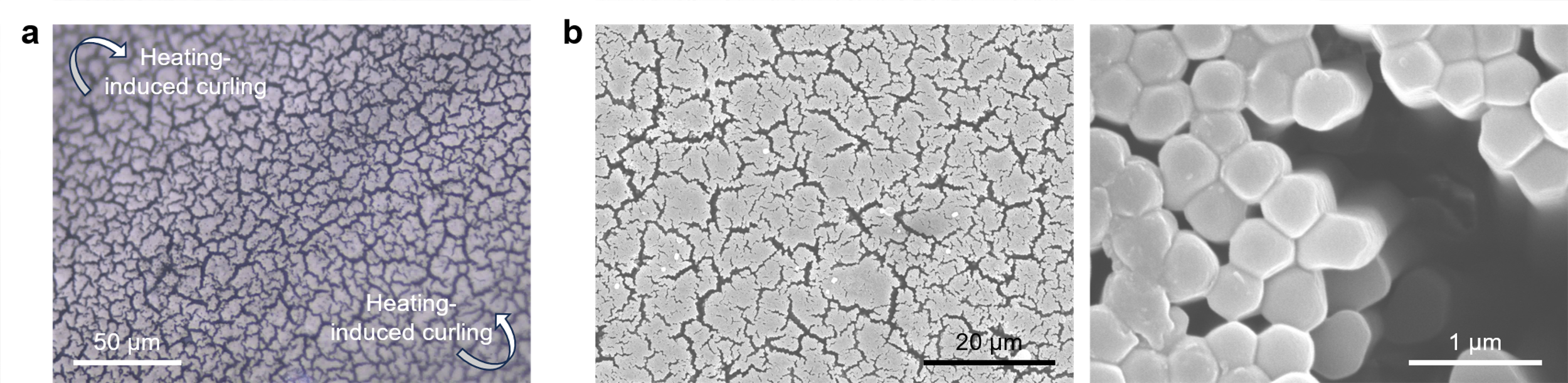


**Figure S2. Stability of LCE nanowires after heating-induced film curling.** **a**, Polarized light micrograph (reflection mode) of LCE films with 400 nm-diameter nanowires at 80°C. **b**, SEM images of 400 nm-diameter nanowires on LCE film after heat-induced curling and subsequent cooling to room temperature. These results demonstrate the preservation of nanowire assembly structure following thermal deformation cycles.


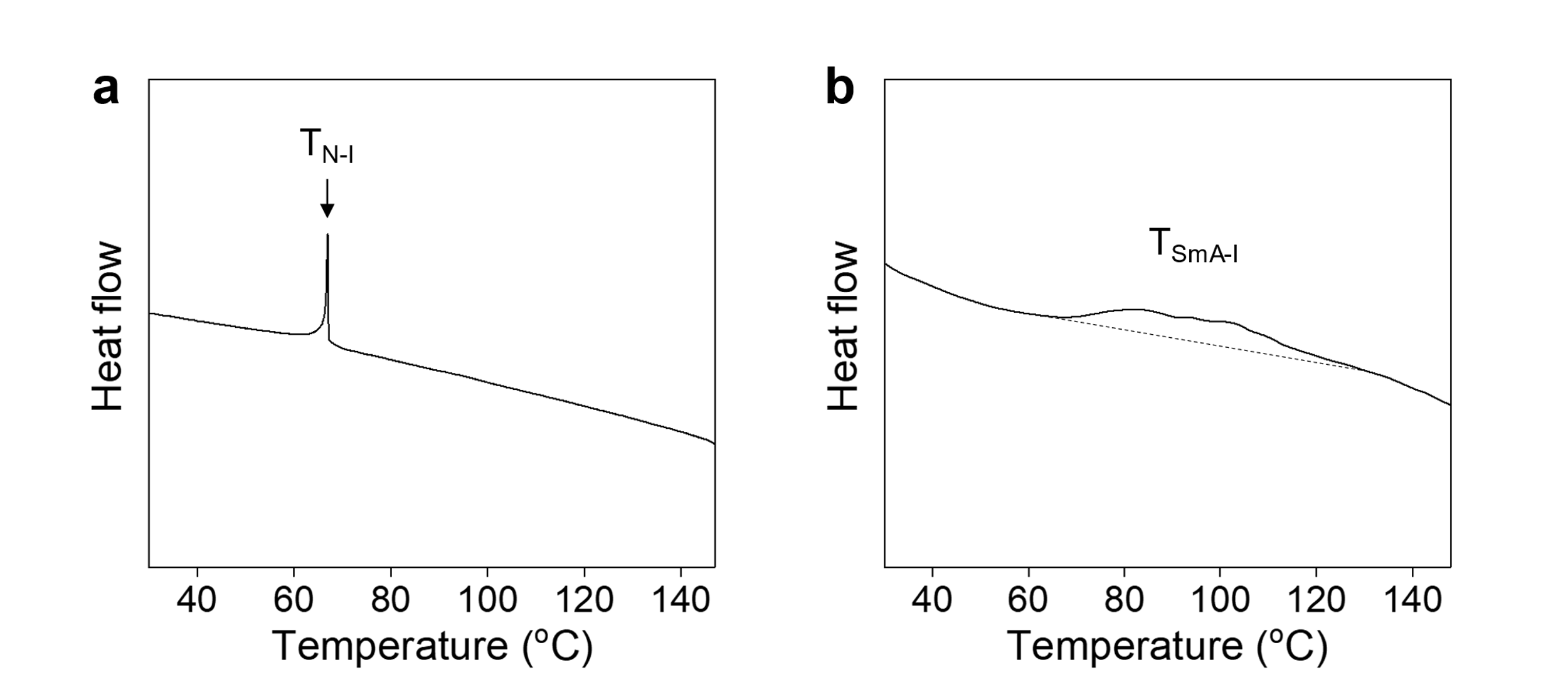


**Figure S3. Thermal characterization of LC mixtures and LCE nanowire films.** **a**, DSC heat flow of a mixture containing 90 wt% end-on LC monomer and 10 wt% RM257 crosslinker. **b**, DSC heat flow of the polymerized LCE nanowire film synthesized from the same mixture composition after an additional 1 wt% DMPAP photoinitiator was added. The broad transition temperature of the LCE nanowire film matches with the observed thermal-induced deformation.


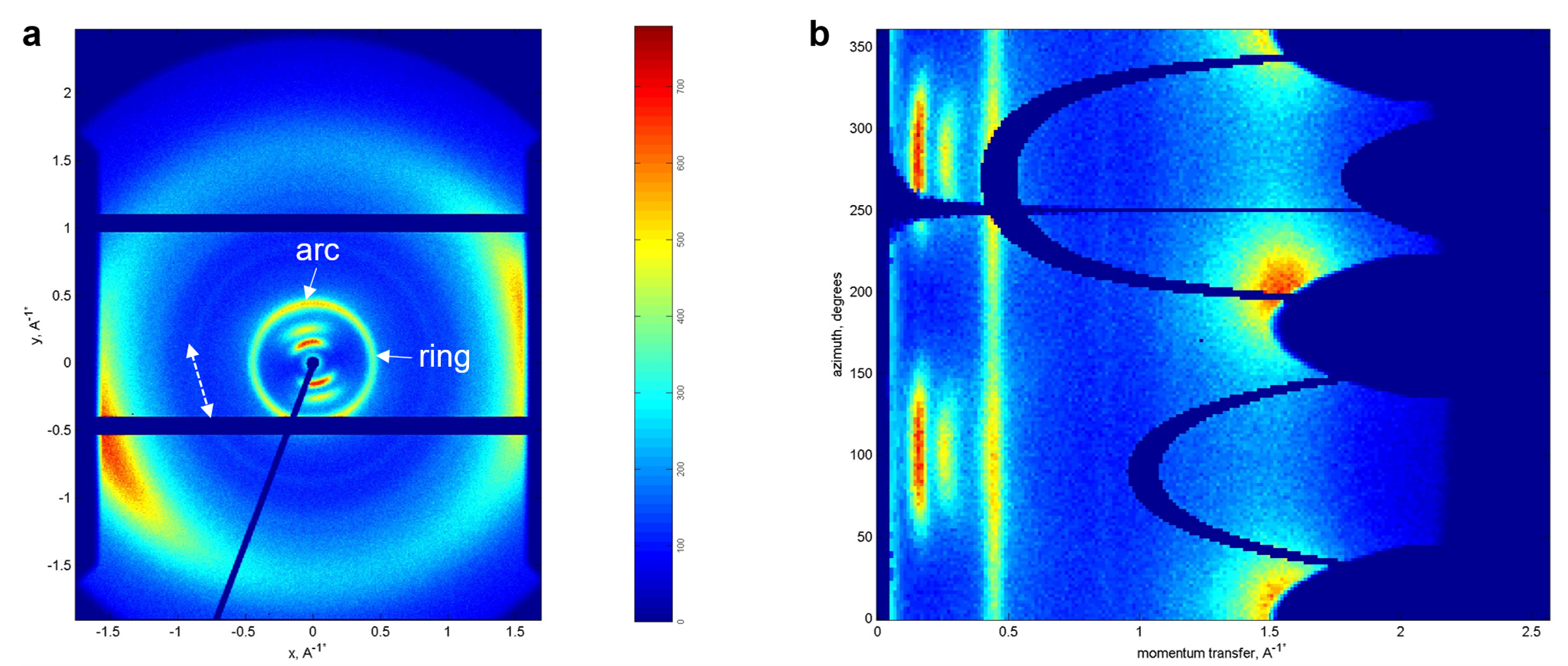


**Figure S4. X-ray scattering characterization of LCE films after nanowire removal.** 2D X-ray scattering patterns of LCE films after nanowire removal as a function of (**a**) azimuthal angle and (**b**) momentum transfer. The white, double-headed arrow indicates the rubbing direction of polyimide-coated glass slide during LCE synthesis.


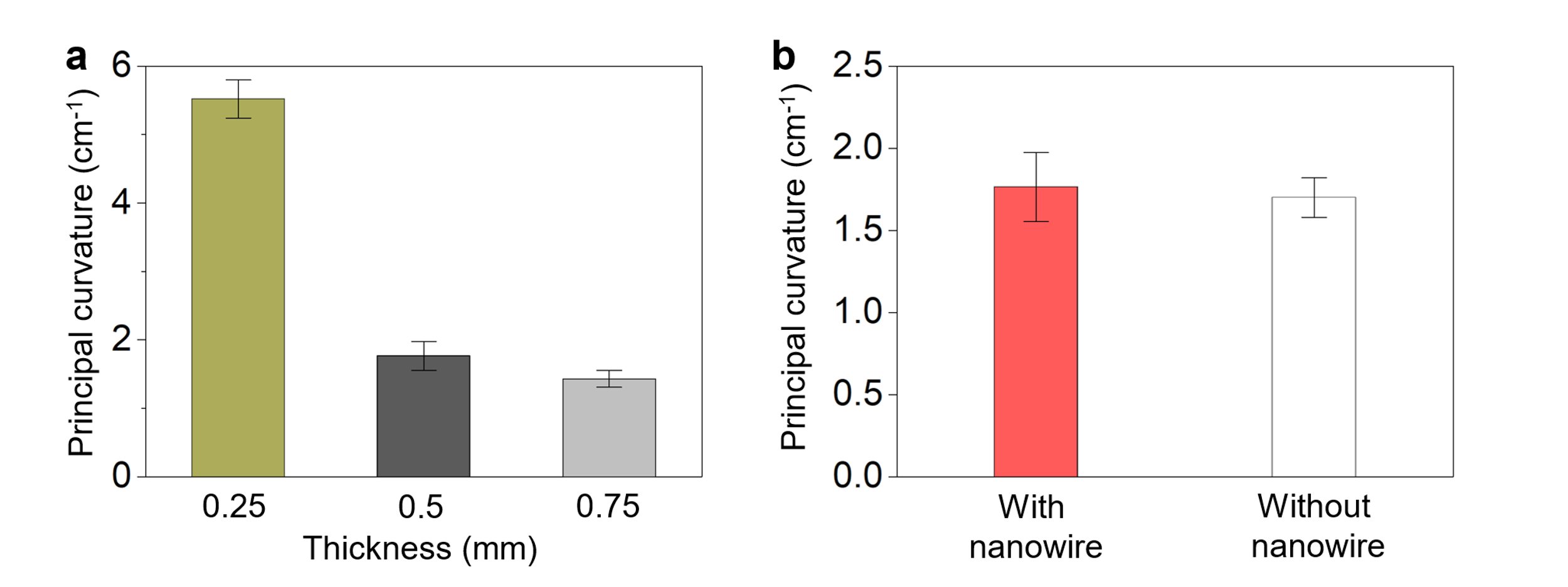


**Figure S5. Effect of film thickness and nanowire structure on the deformation of LCE films.** **a**, Principal curvature of LCE nanowire films as a function of the LCE base film itself, not the length of the nanowires. **b**, Comparison of principal curvature of 0.5 mm-thick LCE films with and without nanowires.


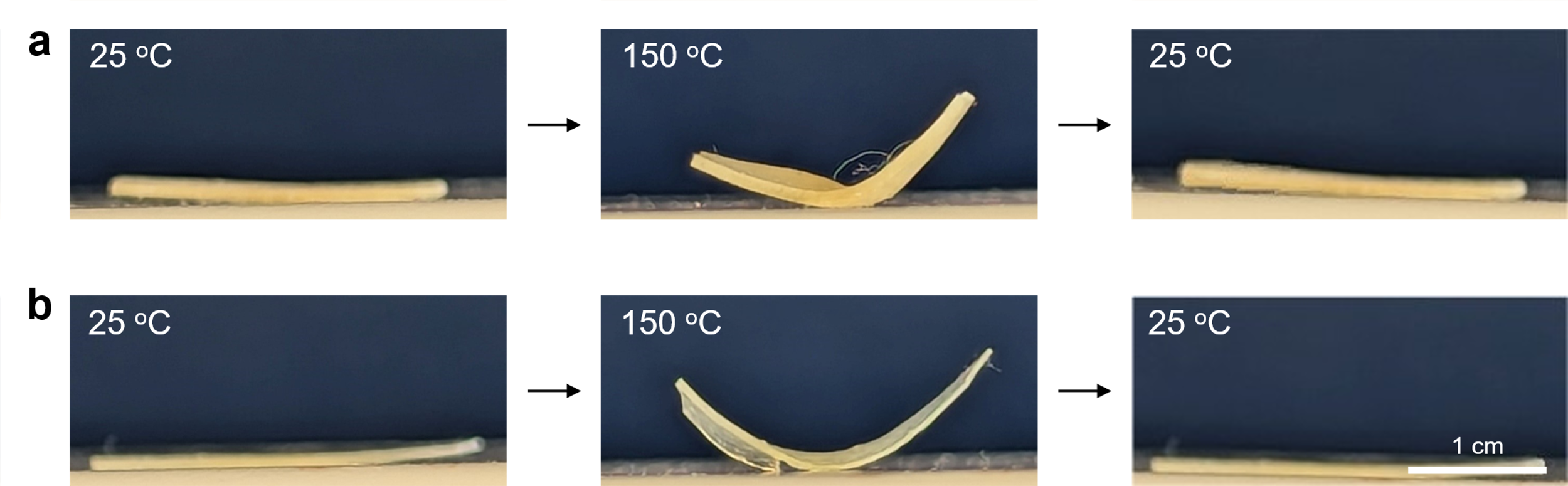


**Figure S6. Shape deformations of LCE films made without nanowire structures and after removal of the nanowire structures.** **a,** Thermal-induced curling of an LCE film after the removal of nanowires. **b,** Thermal-induced curling deformation of a flat LCE film fabricated using a DMOAP-functionalized glass slide and a polyimide-coated glass slide, without the use of an AAO template.


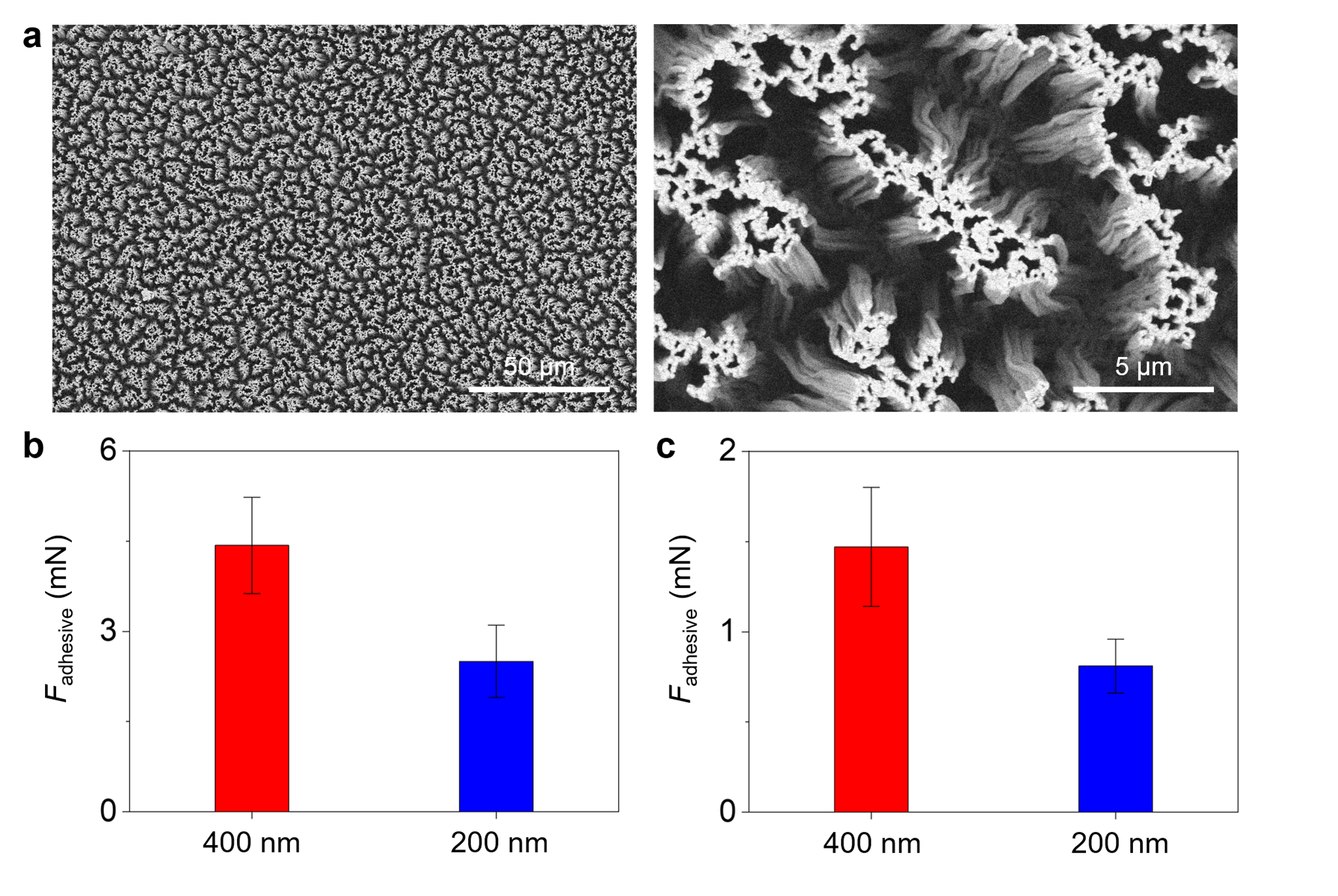


**Figure S7. Effect of nanowire diameter on the adhesion properties of LCE nanowire films.** **a**, SEM images of the 200 nm-diameter nanowires on the LCE film. **b**, Solid adhesive forces for LCE nanowire films with 200 nm and 400 nm-diameter nanowires on flat glass. A 20 kPa pressure was applied for 3 seconds before measuring the adhesive force. **c**, Liquid adhesion force of water droplets on vertical LCE nanowire films with 200 nm and 400 nm-diameter nanowires. Adhesion force was determined by the volume of water lifted by the LCE nanowire film.


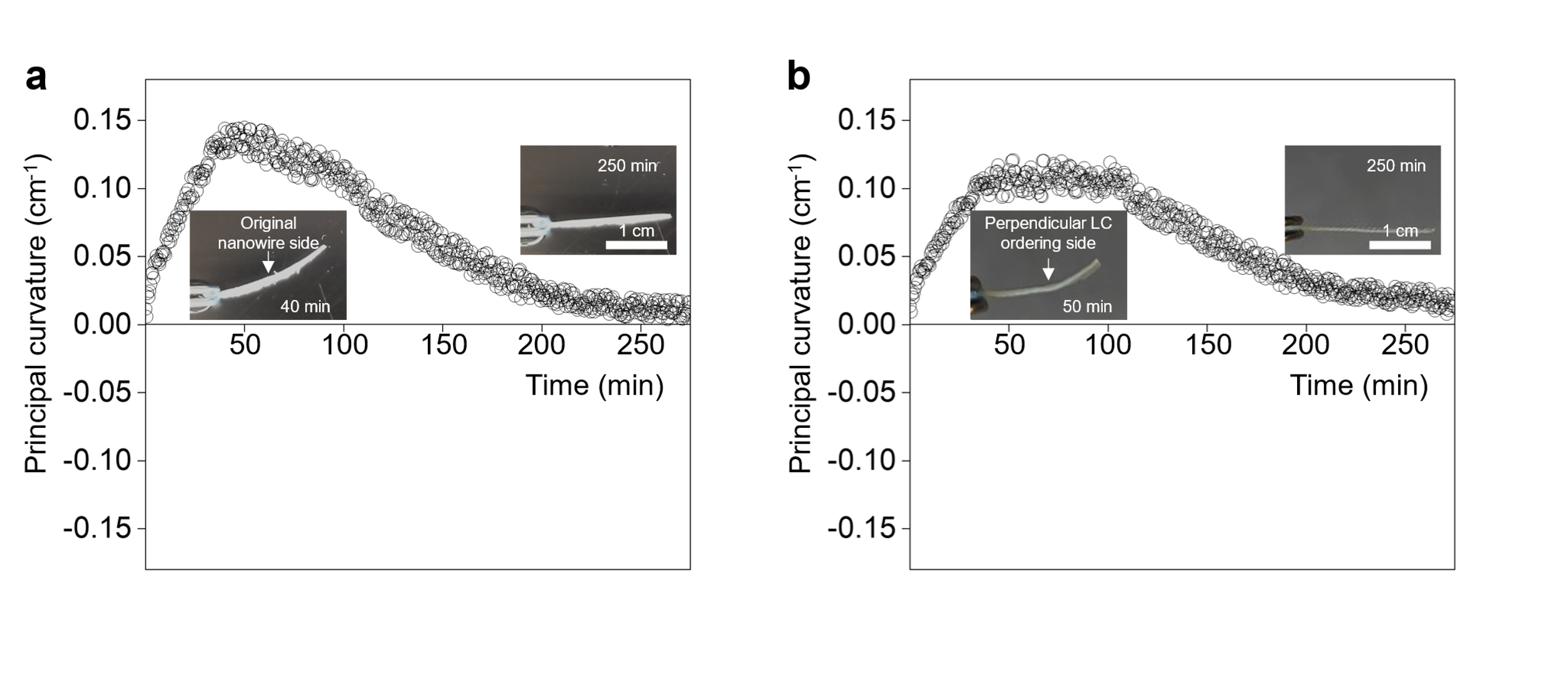


**Figure S8. Toluene droplet-activated deformation of LCE films made without nanowire structures and after removal of the nanowire structures. a,** Plot and accompanying images of the deformation of a nanowire film after removal of the nanowires in response to a 40 µL droplet of toluene placed on the surface. As can be seen, the film still curls in the direction of the side that would have had nanowires on the surface, which is the opposite observation as the films that still have nanowire surface structures. **b,** Plot and accompanying images of the deformation of an LCE made without nanowires but with the same surface alignments. Again, the film curls towards what would be the nanowire side of the film.


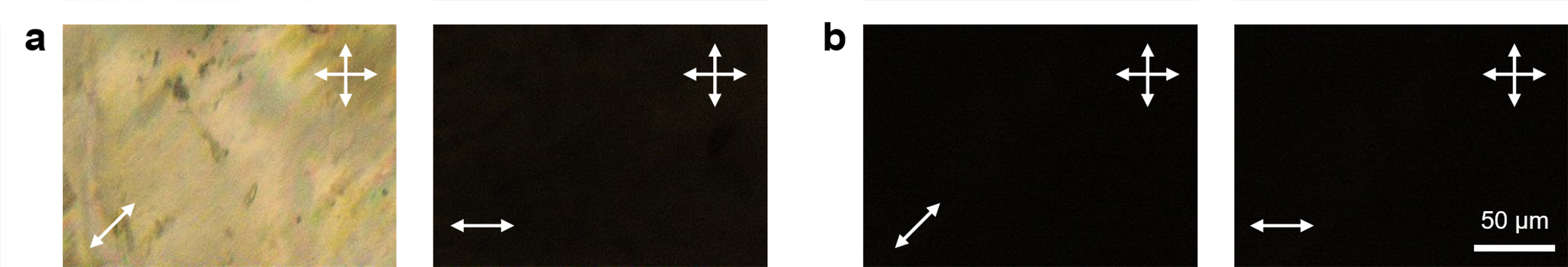


**Figure S9. Polarized light microscope images of LCE films immersed in toluene.** (**a**) Dry and (**b**) swollen flat LCE film after immersion in toluene upon a rotation between crossed polarizers. The flat LCE film was fabricated using a DMOAP-functionalized glass slide and a polyimide-coated glass slide, without the use of an AAO template. The disappearance of the bright to dark transition points towards the swelling and disorganization of the LCs.


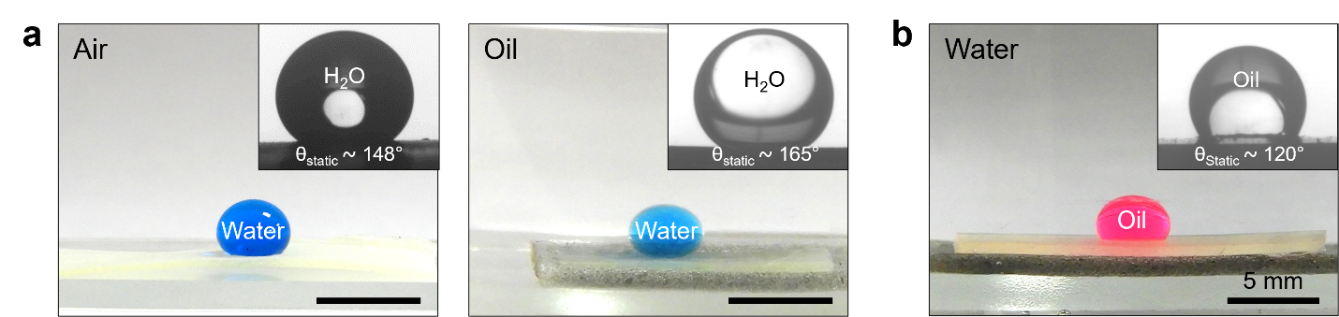


**Figure S10. Effect of chemical modification on the wettability of LCE films. a**, Photographs and contact angle goniometer images (inset) of water droplets on unmodified LCE nanowire films in air and dichloromethane oil. **b**, Photograph and contact angle goniometer image (inset) of a dichloromethane droplet on a chemically modified LCE film without nanowires underwater. The volume of all liquid droplets is 5 μL


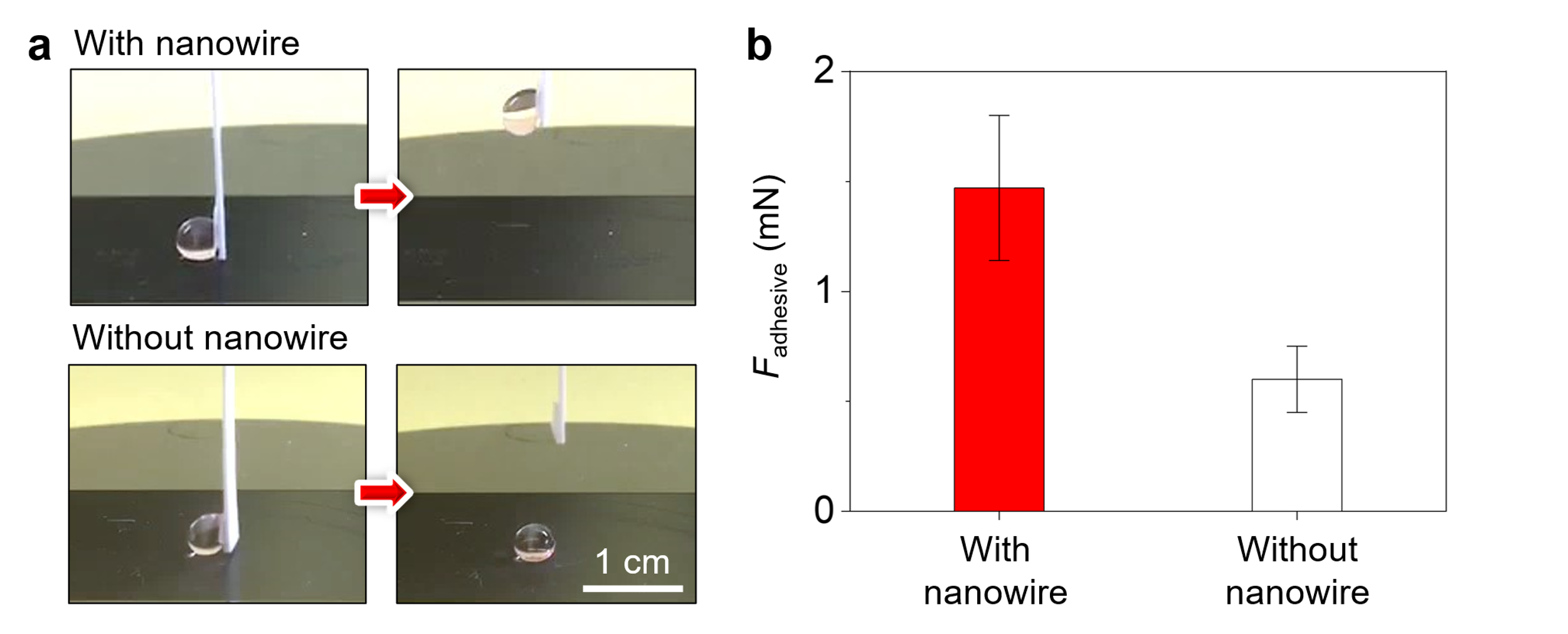


**Figure S11. Adhesion of water droplets to LCE** **nanowire films.** **a,** Photographs and **b,** measured adhesion force of water droplets on vertical LCE films with and without nanowires. A 3 mm-wide LCE film was gently pressed against a 15 μL water droplet before being lifted. Adhesion force was determined by the volume of water lifted by the film.


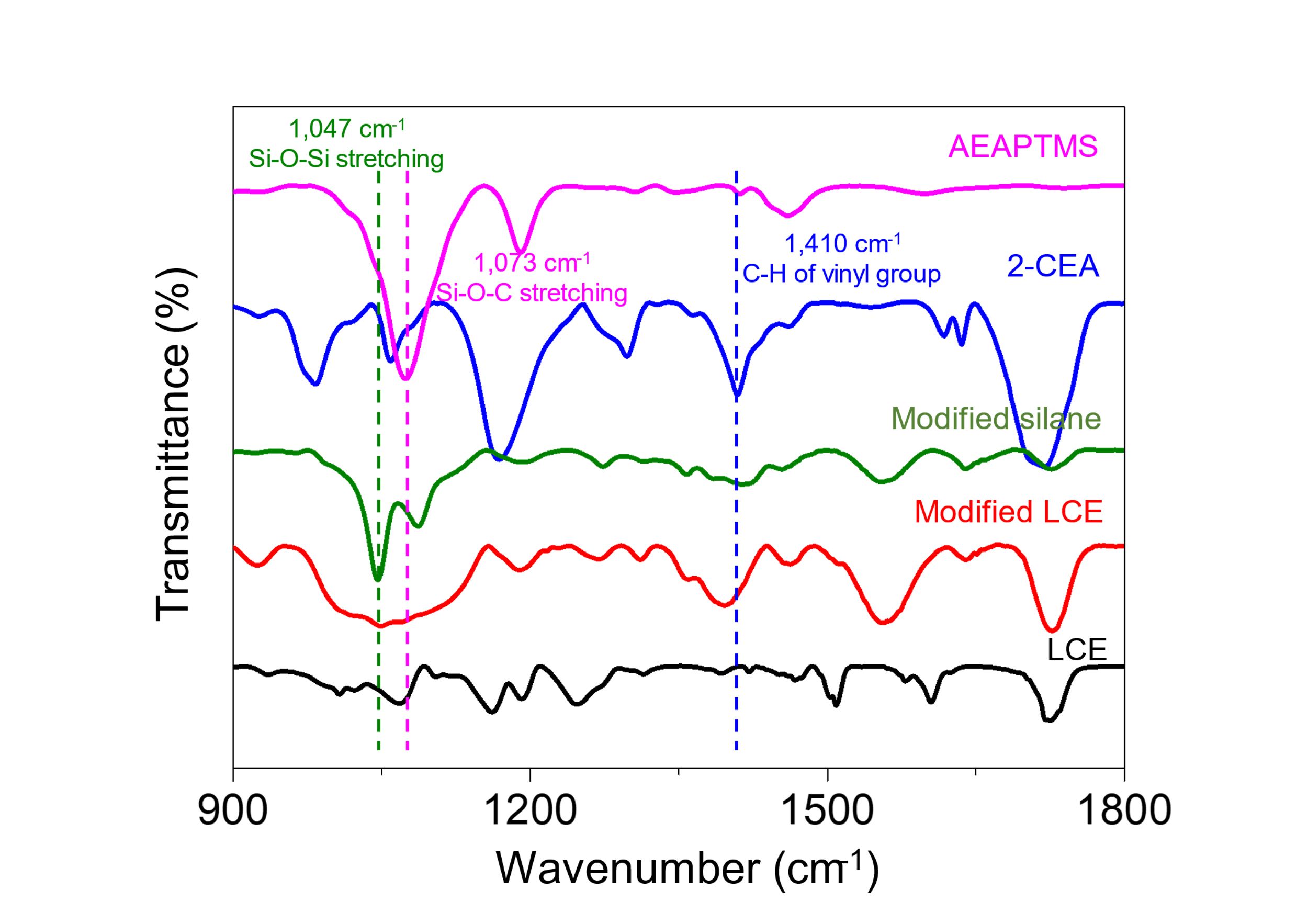


**Figure S12. ATR-FTIR analysis of surface chemical modification of LCE films.** The spectra confirm silane self-condensation and the 1,4-conjugate addition reaction between acrylates and amines. The Si−O−Si stretching peak at 1,047 cm^−1^ indicates AEAPTMS condensation, while the disappearance of the 1,410 cm^−1^ peak verifies the acrylate−amine reaction.


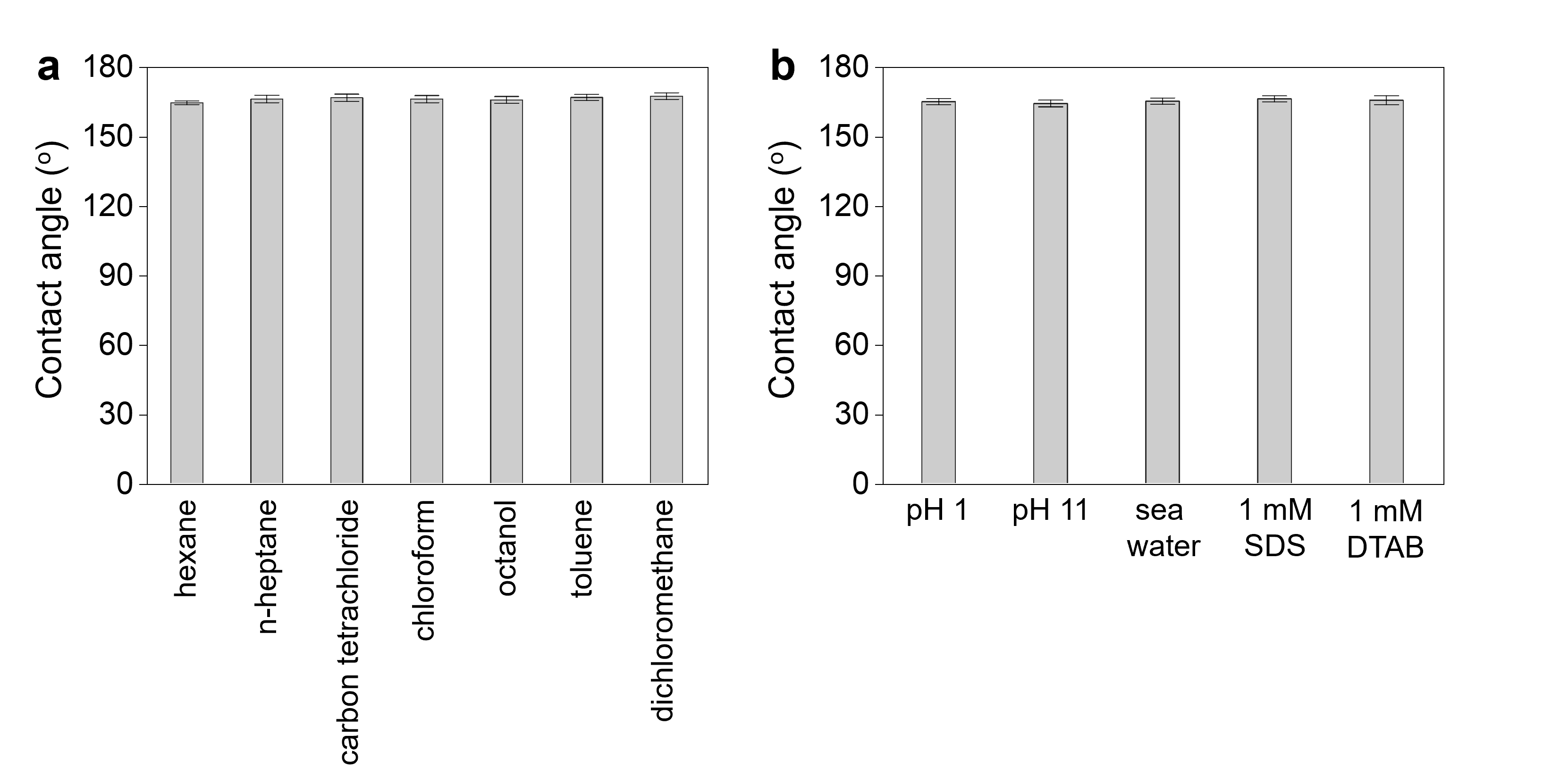


**Figure S13. Static contact angles of oil droplets on chemically modified LCE films. a**, Contact angles of various oils with different surface tensions on modified LCE nanowire films underwater. The surface tensions are: hexane (17.91 mN/m), n-heptane (20.14 mN/m), carbon tetrachloride (26.43 mN/m), chloroform (26.67 mN/m), octanol (27.60 mN/m), toluene (28.53 mN/m), and dichloromethane (33.30 mN/m). **b**, Contact angles of dichloromethane on modified LCE nanowire films in different aqueous environments: pH 1, pH 11, artificial seawater, 1 mM SDS, and 1 mM DTAB after 30 days.


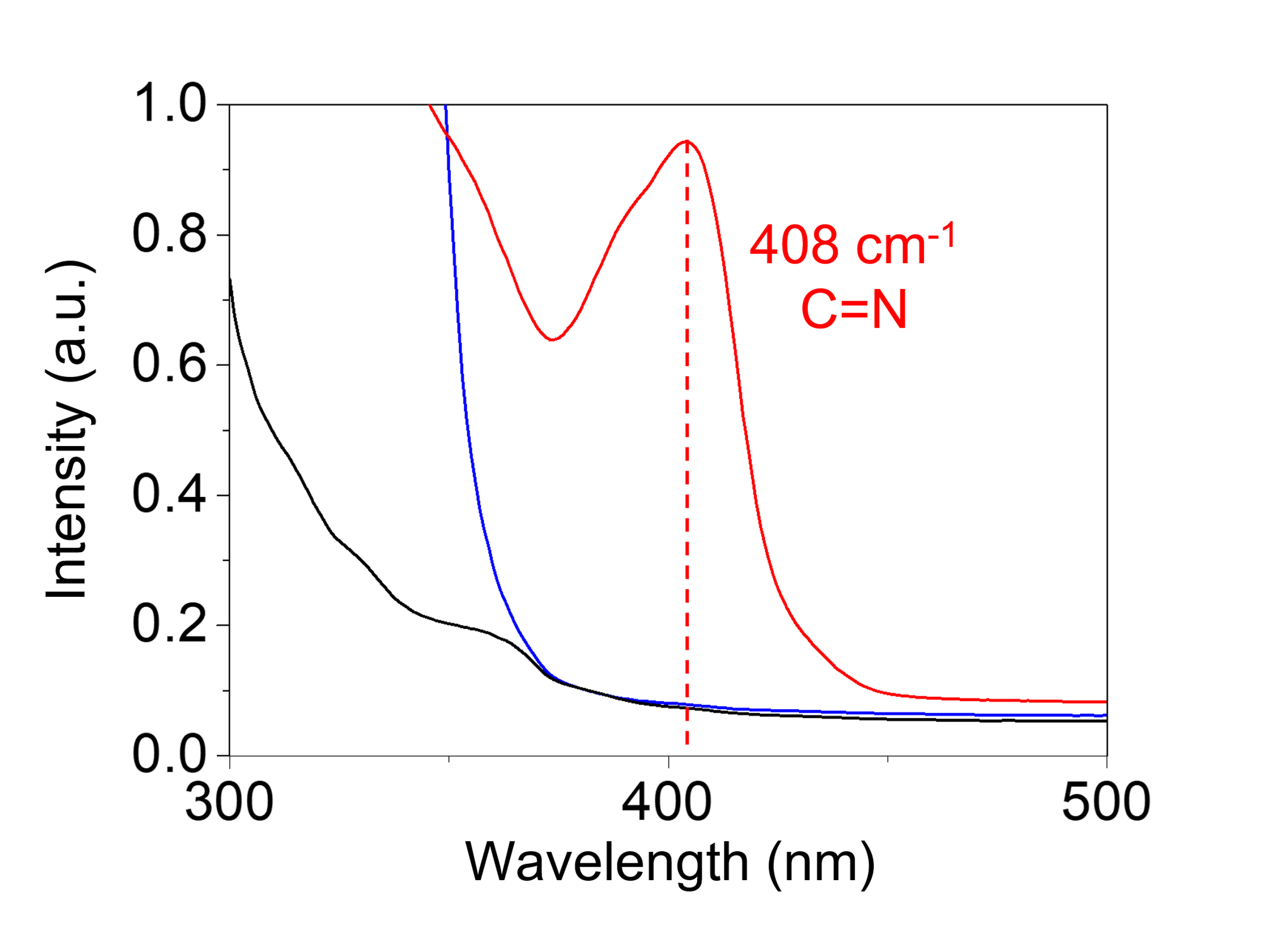


**Figure S14. UV-visible spectroscopic analysis of the reaction between vanillin and decylamine.** The black and blue lines represent vanillin and decylamine, respectively. The red line shows the formation of 4-((decylimino)methyl)-2-methoxyphenol, with the peak at 408 nm indicating the imine bond formed during the reaction between vanillin and decylamine.


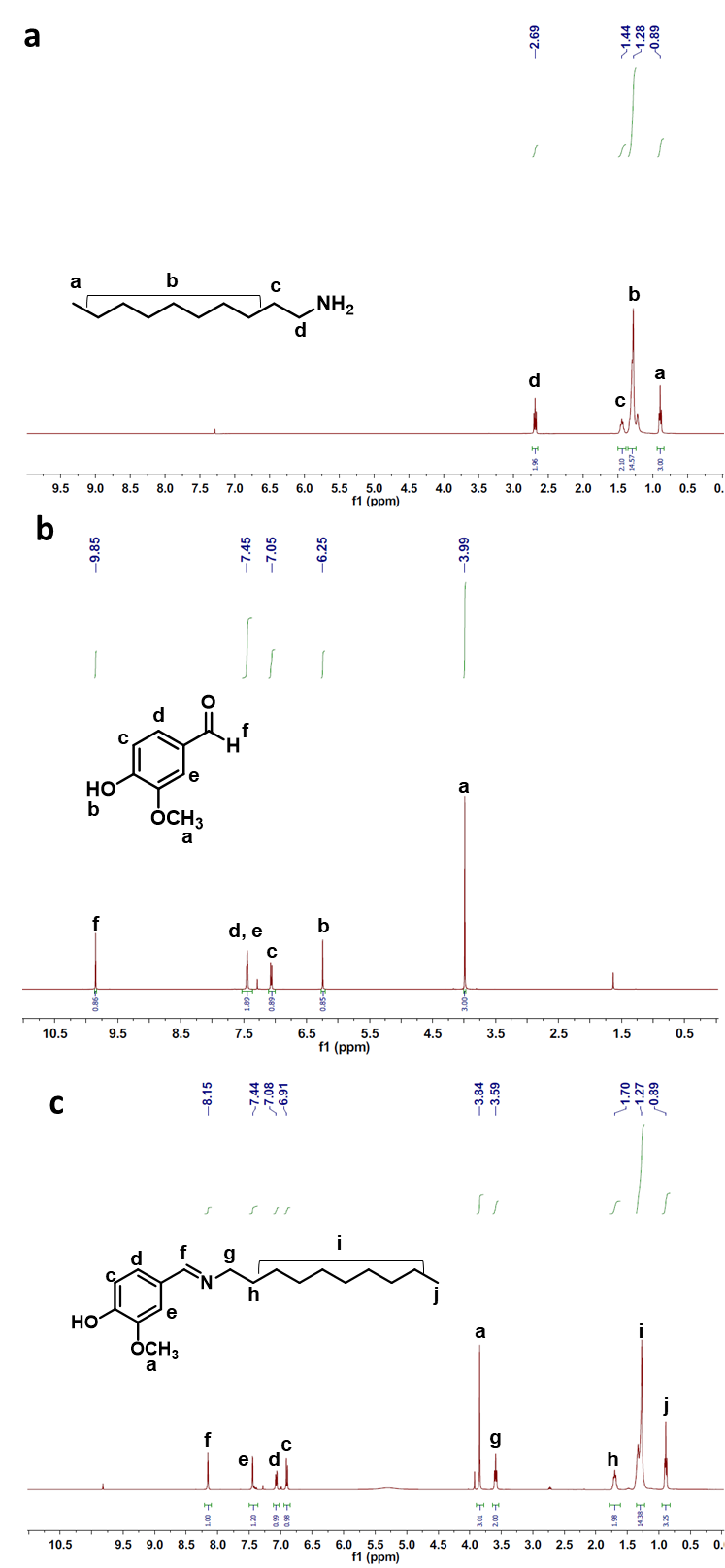


**Figure S15.** **^1^H NMR analysis of the reaction between vanillin and decylamine.** ^1^H NMR spectra of **a,** decylamine, **b,** vanillin, and **c,** 4-((decylimino)methyl)-2-methoxyphenol, produced by mixing droplets of decylamine and vanillin using LCE nanowire film-based droplet mixers.


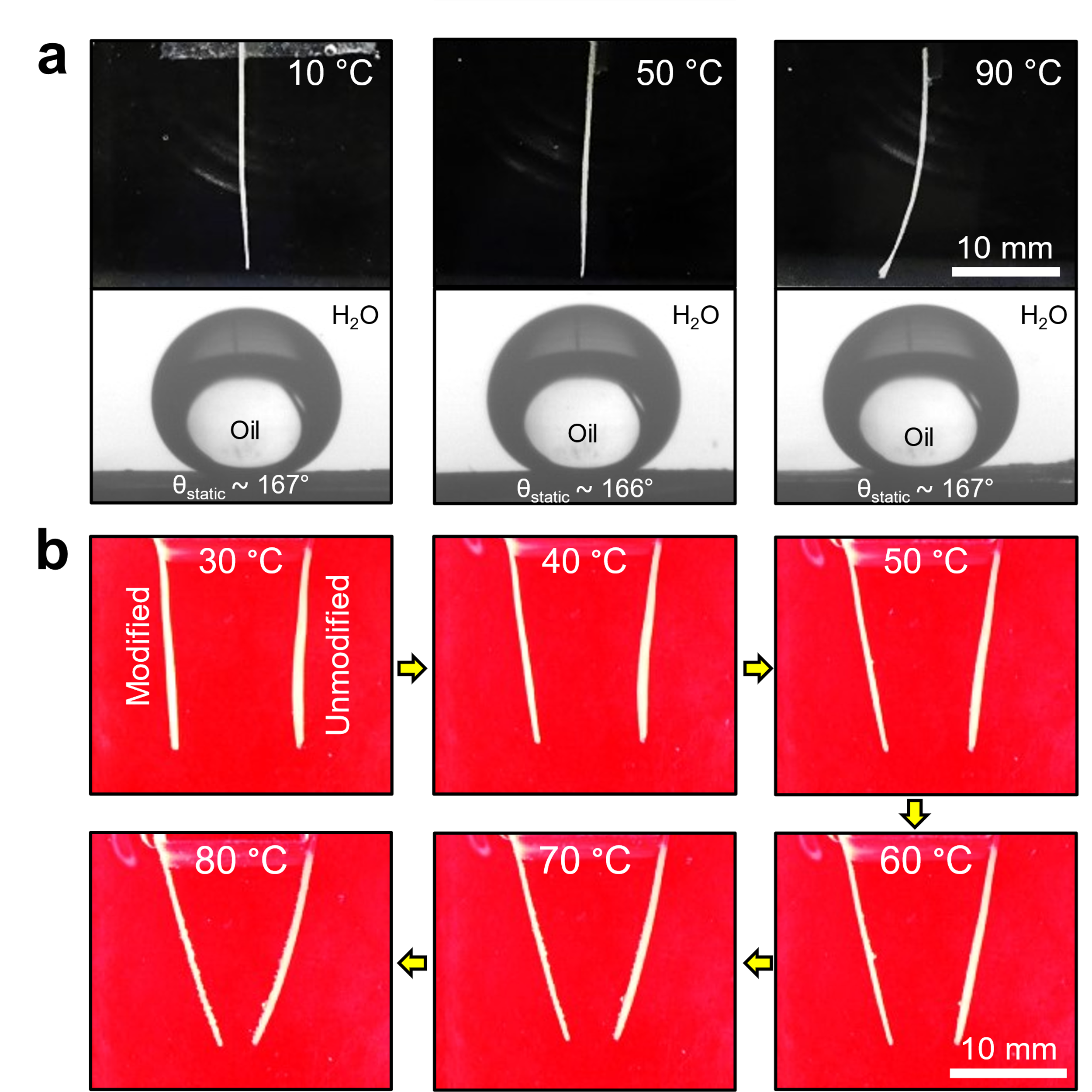


**Figure S16. Reversible shape deformations of LCE nanowire films and their effect on oil wettability underwater.** **a**, Photographs of the shape deformation of chemically modified LCE nanowire films underwater as a function of temperature and the corresponding contact angle goniometer images of dichloromethane on the LCE films. **b**, Photographs showing the shape deformation of LCE films with and without surface chemical modification when exposed to IR light from a distance of 10 cm.


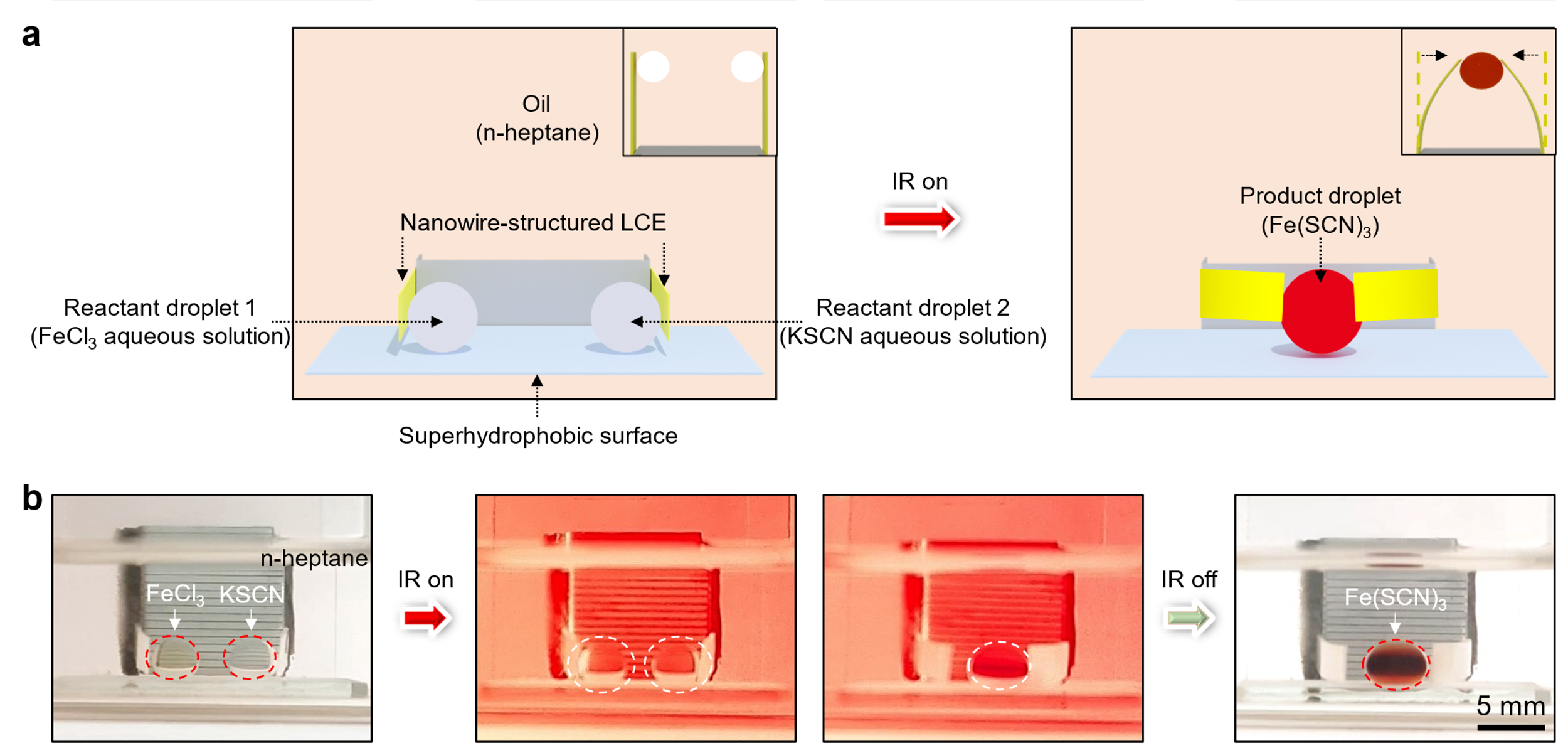


**Figure S17. LCE nanowire film-based droplet mixer triggers chemical reactions in water droplets under oil. a,** Schematic of the experimental setup and **b** photographs show water droplets (10 μL) containing FeCl_3_ (left) and KSCN (right) placed on a superhydrophobic surface beneath n-heptane oil. Upon IR light exposure (15 min), the actuation of LCE nanowire arms (fixed at the sides) mixes the droplets, resulting in the formation of a blood-red colored product, Fe(SCN)_3_.

**References**

[1] a) Z. Wang, Z. Wang, Y. Zheng, Q. He, Y. Wang, S. Cai, *Sci. Adv.* **2020**, 6, eabc0034; b) Y. Zhao, Y. Chi, Y. Hong, Y. Li, S. Yang, J. Yin, *Proc. Natl. Acad. Sci.* **2022**, 119, e2200265119.

[2] K. Maji, U. Manna, *J. Mater. Chem. A* **2018**, 6, 6642.

[3] A. Borbora, R. L. Dupont, Y. Xu, X. Wang, U. Manna, *Mater. Horiz*. **2022**, 9, 991.

[4] a) Y. Xu, R. L. Dupont, Y. Yao, M. Zhang, J.-C. Fang, X. Wang, *Macromolecules* **2021**, 54, 5376; b) Y. Yao, A. M. Wilborn, B. Lemaire, F. Trigka, F. Stricker, A. H. Weible, S. Li, R. K. A. Bennett, T. C. Cheung, A. Grinthal, M. Zhernenkov, G. Freychet, P. Wasik, B. Kozinsky, M. M. Lerch, X. Wang, J. Aizenberg, *Science* **2024**, 386, 1161.

[5] K. L. Johnson, K. Kendall, A. D. Roberts, *Proc. R. Soc. London A.* **1971**, 324, 301.

[6] Y. Yao, R. K. A. Bennett, Y. Xu, A. M. Rather, S. Li, T. C. Cheung, A. Bhanji, M. J. Kreder, D. Daniel, S. Adera, J. Aizenberg, X. Wang, *Proc. Natl. Acad. Sci.* **2022**, 119, e2211042119.
